# What Counts in General Chemistry? An Exploratory Look at What Was Emphasized and Rewarded on General Chemistry Exams Across the United States

**DOI:** 10.1021/acs.jchemed.6c00309

**Published:** 2026-07-05

**Authors:** Nicole C. Greco, Niall J. Ellias, Ryan L. Stowe

**Affiliations:** Department of Chemistry, University of Wisconsin—Madison, Madison, Wisconsin 53706, United States

**Keywords:** First-Year Undergraduate/General, Chemical Education Research, Assessment, Professional Development

## Abstract

General chemistry courses are prerequisites for many Science, Technology, Engineering, and Mathematics (STEM) majors. This reflects a, perhaps tacit, assumption that performance in introductory chemistry relates to one’s suitability for a STEM career. Unfortunately, our field has little idea what is required to succeed in general chemistry courses across the United States. Therefore, we cannot evaluate if general chemistry grades are an appropriate proxy for one’s potential in STEM. Here, we describe the intellectual work emphasized and rewarded on 235 exams administered in general chemistry courses at 57 colleges/universities across the USA. We parsed assessments into *items*, which might consist of multiple *subparts*, each with at least one embedded *task*. We focus on exams as they are ubiquitous in general chemistry courses and typically consequential for students’ grades. First, we used the 3-Dimensional Learning Assessment Protocol to describe exam points allotted to items that prompt students to use core disciplinary ideas to make sense of phenomena (i.e., 3D Learning). Findings showed that prompting for 3D Learning on assessments was rare regardless of institution type, comprising less than 7% of exam points in our data set. Next, we developed and applied a protocol to describe tasks that did not have the potential to engage students in using core ideas in science practices (i.e., 0D *tasks*). This revealed that rote calculations were overemphasized comprising over 27% of all 0D *tasks*. Findings indicate that general chemistry courses gate access to STEM by student facility with rote calculations and drawing/labeling specialist pictures. We unpack why this is problematic and provide recommendations for building chemistry assessments that have the potential to be more meaningful and equitable.

## Graphical abstract

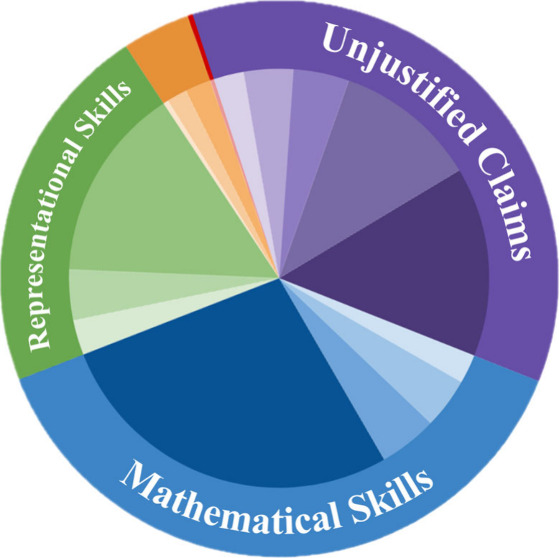

## Introduction

Whether students succeed in introductory (i.e., general) chemistry powerfully impacts their prospects of earning a degree in Science, Technology, Engineering, or Mathematics (STEM), which in turn affects whether they can pursue a science or science-adjacent career.^1^ Large-scale empirical studies have demonstrated that students whose identities are marginalized are often overrepresented among those who fail general chemistry.^1–3^ This course thus contributes to underrepresentation of Black and Brown students in STEM. Progress toward the goals of cultivating a diverse STEM workforce requires that we transform general chemistry from gatekeeper to gateway.

Who succeeds in general chemistry is powerfully affected by what is emphasized and rewarded on course assessments, particularly high-stakes assessments. A recent study by Gibbons and colleagues demonstrated that more than 90% of surveyed instructors administered exams.^4^ Prior work conducted by our group suggests these exams are often consequential to students’ grades. General chemistry enactments we characterized as part of a cross-sectional study dedicated between 45% and 65% of course points to high stakes exams (i.e., midterm and final exams).^5,6^ The fact that assessments function to define what success means in a course is not lost on students. The types of performances emphasized and rewarded on quizzes and exams send powerful implicit messages to students about the nature of knowledge and learning in the course (and in the discipline).^7–13^ This is all to say that those of us invested in improving college STEM education need to care about assessments; the students certainly do, as their grades depend on them.

There is reason for concern about the state of general chemistry assessments nationwide. Small-scale studies that characterized the performances emphasized and rewarded on such assessments have found that most prompts emphasize performance of disaggregated skills and recall of facts.^5,6,14–16^ This is problematic for (at least) two reasons: (1) exams that place undue emphasis on rote calculations may act to marginalize students on the basis of their access to precollege math preparation,^6,17^ and (2) skill- and fact-focused exams misrepresent the intellectual heart of chemistry (i.e., making sense of phenomena via use of molecular models).^18^ The role of math-focused chemistry tasks in perpetuating inequity has led several scholars to hypothesize that the status quo assessment emphasis in general chemistry is a central reason to why students whose identities are marginalized are more likely to fail this course.^6,17^ Importantly, this finding is due to inequitable access to precollege math preparation, not minoritized students being inherently worse at math.^19,20^

Unfortunately, we know very little about the sorts of performances commonly emphasized on general chemistry exams nationwide. That is, we do not know whether our observation that a small number of institutions emphasize mostly recall and mathematical skills in general chemistry holds across a larger sample. *This is critical information*. If, across the country, success in general chemistry means execution of rote skills and recall of facts, then changing what we assess becomes a necessary—though likely not sufficient—step toward productive reform. Here, we describe a more complete picture of what counts (i.e., earns credit) in general chemistry. The following research question guided this study:

- What types of performances are emphasized and rewarded across a nationwide sample of general chemistry exams?

While we do not claim this work is comprehensive, this study (to our knowledge) represents exploration of the largest general chemistry exam database yet assembled. The exams we describe here played a role in demarcating the possible futures for tens of thousands of students. To contextualize our work, we briefly review literature that demonstrates the importance of assessments as sorting tools and as powerful mediums for communicating what counts in a class. We then explore efforts our community has undertaken to shift what is assessed in general chemistry. The empirical study that follows thus provides insight into the extent to which these reforms have shifted the assessment practices of the national chemistry education community.

## Prior Research and Conceptual Framework

### Assessments Matter

There are two main ways in which assessments are consequential to students: (1) performance on assessments is used to rank and order students and thereby gate who can continue in STEM and who cannot, and (2) assessments communicate consequential messages about what knowledge and ways of knowing have value.^21^ We will consider each in turn.

For most college chemistry students, success in the course means high grades on course-embedded exams. Two strands of evidence support this claim. First, a large-scale study of instructors’ assessment practices teaches us that virtually everyone gives exams (98.3% give midterms and 92.6% give final exams.)^4,22^ Second, smaller-scale work demonstrates that exam scores make up a large portion of students’ course grade.^14^ Given that one must pass general chemistry to earn most STEM majors and pursue many STEM careers, it is reasonable to say students’ exam performance in this class has a direct and material impact on their possible futures. Failing general chemistry can (and does) prevent many students from pursuing their chosen vocation.^1^

The role of assessments in establishing what constitutes success in a course means that assessments also impact *who* is likely to succeed. We and others have reported that general chemistry items which emphasize rote calculations are inequitable to students on the basis of their access to prior math preparation.^6,17^ Due to inequities baked into the structure of K–12 education, individuals with less access to high-quality mathematics preparation tend to belong to groups marginalized by dominant policies and practices in society.^19,20,23–27^ Stated succinctly, placing undue emphasis on calculations is one way in which college STEM ecosystems marginalize students with certain socially constructed identities (i.e., Black, Indigenous, and Latine students). Note that we are *not* claiming all math should be removed from general chemistry; there are certainly many important phenomena that require mathematical thinking to understand (e.g., using product energies to determine whether the relative abundance of products is consistent with a reaction that has reached equilibrium). Instead, we urge our community to attend to who benefits and who is harmed by emphasizing particular mathematical skills in certain ways at certain times in the course.

In addition to determining who is permitted to pursue a STEM major or career, exams also communicate what knowledge and ways of knowing have value in a course. For example, an exam in which students must draw pictures that match an instructor-authored answer key for credit may communicate “knowledge is justified by aligning with the answer key” and “good knowledge must have a particular structure.” Schwarz and colleagues^8^ demonstrated that aggregated exam-embedded messages about useful ways to know and learn (i.e., epistemological messages^21^) may synthesize to give more macro-level understandings of knowing and learning in the course. Specifically, they observed that students enrolled in a transformed organic chemistry course increasingly saw their task as constructing knowledge products that matched the answer key. Other potentially more useful ways of constructing and justifying knowledge (e.g., justifying claims by seeking alignment with evidence) were not experienced as useful in the focal course. Given that many of the reasons we say students should take chemistry are about supporting useful ways of thinking (vs learning useful content^28^), we should care about what our exams tell students about valued knowledge products and processes.

### What Should We Assess?

We expect the statement “exams matter” will be relatively uncontroversial to most readers. After all, scholars have been urging us to refine our exams for at least 70 years.^29^ More recently, members of the chemistry education community have carefully articulated what sorts of performances are worth emphasizing and rewarding on written assessments. Some have called for increased emphasis on “conceptual” tasks and a corresponding decrease of “algorithmic” exercises.^30,31^ Others have advocated for our tests to include more prompts that require higher-order cognitive skills such as problem solving, decision making, and critical thinking.^32^ Unfortunately, due in part to the lack of consensus around what, exactly, is meant by terms like “conceptual understanding” and “critical thinking,” ^33,34^ these exhortations have been challenging for many to put into practice.

Over the past decade, substantial efforts have been underway to build more nuanced and actionable understandings of what should be assessed in chemistry class. The language of several such efforts is derived from the National Academies’ *Framework for K–12 Science Education*,^35^ which envisions science learning as a process of making sense of phenomena via leveraging fundamental disciplinary ideas in scientific knowledge building practices.^36–41^ The notion that science learning involves using disciplinary core ideas in science practices as framed by crosscutting concepts is referred to as “3-Dimensional Learning” in the chemistry and science education literature. 3D performances, it is claimed, better represent real scientific work and so should be emphasized and rewarded on high- and low-stakes assessments.^42^ This claim is supported by the grounding of the K–12 *Framework* in studies of professional scientists constructing, refining, and communicating knowledge in communities.^43,44^

But what makes an assessment item “3D?” While we acknowledge this can be a complex question if we are after evidence that a particular assessment item is experienced by students as an opportunity for sensemaking (vs answermaking), we (and many others) can make relatively straightforward claims about whether an item *has the potential to* elicit evidence of 3D Learning. Doing so involved characterizing assessment items using a protocol developed by Laverty and colleagues called the 3-Dimensional Learning Assessment Protocol (or 3D-LAP).^45^ The 3D-LAP provides criteria an item must meet to potentially elicit evidence of student engagement with a science practice, using core ideas, as framed by crosscutting concepts. 3D-LAP results are typically reported by the percentage of points on a given assessment that have the potential to elicit evidence of 3D Learning. This protocol was developed by an interdisciplinary team of discipline-based education researchers and has been used to characterize the assessment emphasis of a variety of large-enrollment STEM courses.^46–50^

### What Do We Assess?

While many scholars in our field have considered what should (and should not) be assessed in large-enrollment chemistry courses,^5,35,51–53^ we have remarkably little idea of what is emphasized and rewarded on quizzes and exams given across the United States. Our group has reported a small-scale analysis of assessment emphasis as a way of highlighting how little chemistry is sometimes assessed in chemistry courses.^5,14^ However, the data set from this work was derived from three relatively homogeneous R1 institutions located in the Midwest. We do not know whether our findings will generalize across a larger sample that includes different types of institutions and different student populations.

The most comprehensive look at assessment emphasis in college STEM we are aware of focused on characterizing the performances on introductory biology exams given by 44 instructors who participated in a professional development workshop.^54^ These instructors represented both faculty and instructional staff and taught at a range of institution types. In this study, Momsen and colleagues found that 93% of the 9713 assessment items reviewed were dedicated to the recall of facts or performance of decontextualized skills (i.e., described by level 1 or 2 of Bloom’s taxonomy^55^). From this analysis, we can infer that students enrolled in the focal classes are likely to take away the message that biology class—and possibly biology as a discipline—is about memorization and regurgitation of facts and rote performance of skills. We do not know how (or whether) the assessment emphasis in college chemistry is similar to what Momsen and colleagues observed for college biology.

If researchers, practitioners, and policymakers are serious about transforming college chemistry learning environments so they are both equitable and meaningful, we must attend to what is emphasized and rewarded on assessments—in addition to many other facets of the learning environment. To do this, we need to have a sense for the current state of general chemistry assessments given throughout the United States.

## Methods

### Data Collection

The research team constructed a list of 200 institutions based on the 2021 U.S. News & World Report’s Top 100 Universities^56^ and Top 100 Liberal Arts Colleges.^57^ The U.S. News & World Report’s rankings were used because top-ranked institutions tend to be well-resourced and have relatively few students transfer prior to completing a four-year degree. We therefore anticipated that such schools would be better able to implement ambitious general chemistry reforms than their less well-resourced peers. This list of institutions was divided among members of the research team, and each researcher compiled a collection of general chemistry instructor emails from their subset of institutions. This yielded a list of 893 chemistry instructor emails. Each of these instructors was provided with study details and asked whether they consent to take part in study activities as approved by the UW–Madison Institutional Review Board (IRB 2021-0451). These activities included describing aspects of their general chemistry course via responding to a survey about course details (e.g., course title, enrollment size, textbook used, relative weight of assessments on course grade) and uploading exam forms.

A total of 117 chemistry instructors consented to participate and completed our survey with 83 of those instructors contributing between one and nine of their exams to our database. For instructors teaching at the same institution, the research team verified the submitted exams were from separate course numbers (e.g., general chemistry I, general chemistry II). Any duplicate courses were compared and only the most information-rich (i.e., folder with complete score breakdowns, more exams) was kept to not overrepresent one course in our findings. This resulted in a data base comprised of 309 exams given by 69 unique instructors from 66 unique institutions. Exams given at primarily undergraduate institutions made up the majority of our database (204 exams from 44 institutions), followed by R1 institutions (83 exams from 17 institutions), and exams given at R2 institutions (22 exams from 5 institutions). Describing assessment emphasis with the 3D-LAP (detailed below) requires that a point breakdown be listed for each assessment item coded as 3-Dimensional. As such, we recontacted instructors whose exams did not have this information to request more detailed item point breakdowns. Few instructors responded with these additional details. Any exams that did not break down the points allotted to 3D items and/or did not list the total points for an exam were excluded from our data set. The analysis described below was carried out on 235 exams from 57 institutions including 36 PUIs (137 exams), 16 R1 universities (76 exams), and 5 R2 universities (22 exams). In total, 4626 assessment *items* were analyzed.

### Data Analysis

The substantial variability in assessment and question formatting (e.g., multiple choice, free response, nested multipart, etc.) across our data set required us to develop a systematic way to define and classify assessment questions regardless of formatting. We refer to this system of classification as an “assessment taxonomy” and present its form in Figure 1.

**Figure 1:**
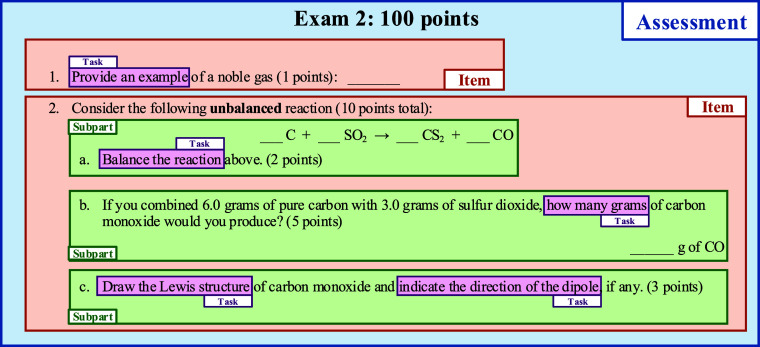
Sample exam excerpt demonstrating how we categorized assessment elements of varying grain sizes.

The largest grain category in this taxonomy is *assessment* (blue box in Figure 1), which is defined as the complete collection of explicitly required prompts worth credit on an exam. Extra credit prompts were excluded from coding as those points were not required by instructors for full credit on the *assessment*. This category represents the 235 exams that were analyzed in our study.

The next-smallest grain size category is the assessment *item*, which is defined as the complete collection of prompts grounded in the same focal context. This is exemplified in Figure 1 as the orange boxes which showcase how Question 1 is a separate assessment *item* from Question 2, but 2a, 2b, and 2c are all related to the same focal reaction and are therefore considered part of the same assessment *item*. This category represents the 4626 assessment *items* analyzed in our study.

An *item* may contain *subparts* which are the next-smallest grain element of our assessment taxonomy. *Subparts* are defined as any distinct assessment prompt contained within an assessment *item*. These are depicted in Figure 1 with the green boxes designating parts a, b, and c, as separate *subparts* of the same assessment *item*, Question 2.

The smallest grain category in our taxonomy is *task*. *Tasks* are defined as prompts for an individual knowledge product (e.g., representation, representation annotations, number, word, or phrase). In Figure 1 (purple box), we can see several *tasks* embedded in *item* 2 *subpart* c—students are to draw a Lewis structure and subsequently label this structure with the direction of the molecular dipole.

### Assessment as an Evidentiary Argument

Assessment is challenging work—one must build activities that surface evidence of student learning and make reasonable inferences from what students write, draw, or say while engaged in these activities. The National Research Council (NRC) refers to this process when they describe approaching “assessment as an evidentiary argument.”^58^ Of course, one cannot know exactly what is going on inside students’ heads when they engage with an assessment in a certain way, so what we consider reasonable inferences from assessment evidence is bounded by how we model learning (what the NRC calls a “model of cognition”). In the present study, we describe assessment emphasis by considering what inferences responses to a given *item*/*subpart* or *task* have the potential to support. For example, a correct response to 2a in Figure 2 would let one reasonably infer that a student could engage in cognitive process­(es) to write chemical reactions—we thus describe this *task* using the code “writing chemical reactions” (described in more detail below). Importantly, we do not claim that the inferences we describe in what follows are the only such inferences a prompt response could support, merely that these seemed reasonable to the authors.

**Figure 2:**
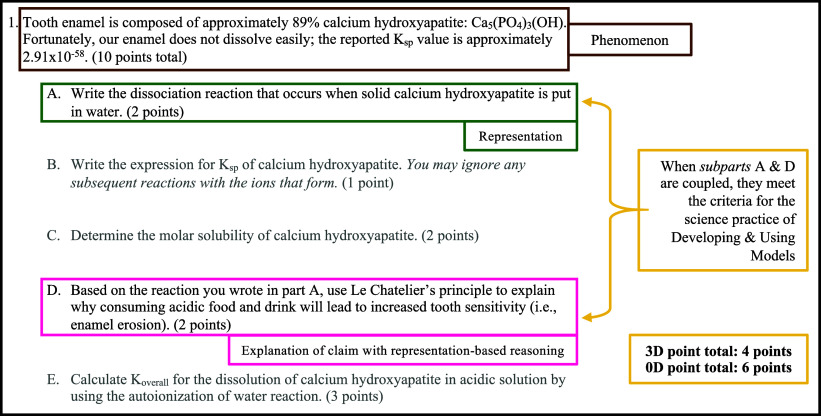
This assessment item includes *subparts* that aggregate to meet 3D-LAP criteria for a science practice and *subparts* that do not. *Subpart* A would not be coded as a 3D question on its own, but when we consider *subparts* A and D together, the 3D-LAP criteria for Developing & Using Models are met. These *subparts* also have the potential to elicit evidence that students are using a core idea (Change & Stability in Chemical Systems) as framed by a crosscutting concept (Stability & Change). Accordingly, points allotted to both *subparts* A and D were categorized as 3D. *Subparts* B, C, and E do not contribute to student engagement in the scientific practice of modeling, nor do they meet the criteria for eliciting evidence of engagement in another science practice, so those points are summed in the 0D category.

Our efforts to describe the intellectual work emphasized and rewarded on the exams in our data set proceeded in two phases. In phase 1, we made use of the 3-Dimensional Learning Assessment Protocol (3D-LAP)^45^ to describe the potential of assessment *items* or *subpart* clusters to elicit evidence of students using fundamental disciplinary ideas to predict, explain, or model chemical phenomena (i.e., 3D Learning). In phase 2, we inductively coded the emphasis of assessment *tasks* that did not meet 3D-LAP criteria for potentially eliciting evidence of 3-Dimensional Learning.

### Phase 1: Analysis of Assessment *Items* with the 3D-LAP

We made use of the 3D-LAP on all assessments in our database to determine whether *items* potentially elicited evidence of students using core ideas to engage in science practices as framed by crosscutting concepts (i.e., 3D Learning). To ensure our use of the 3D-LAP was consistent across the varied exams in our data set, several iterations of inter-rater agreement^59^ were performed, and consensus was reached on all discrepancies. A final Cohen’s Kappa of 0.743 was achieved on approximately 15% of the data set (8 instructors, 36 exams) and the first author then applied the 3D-LAP to the remaining exams. When questions arose about what 3D-LAP codes best described a given *item*, the first author dialogued with the second and third author to reach consensus.

Coding using the 3D-LAP requires first assessing the potential for an *item* to elicit evidence of student engagement in a science practice. An assessment *item* (or *subpart*) would potentially engage students in a science practice if that *item*/*subpart* met all criteria the protocol specified for that practice. As an example, for a constructed response *item* to potentially engage students in developing and using models, that *item* must:

- Give an event, observation, or phenomenon for the student to explain or make a prediction about
- Give a representation or ask the student to construct a representation
- Ask the student to explain or make a prediction about the event, observation, or phenomenon
- Ask the student to provide the reasoning that links the representation to their explanation or prediction

Accordingly, an *item* that prompted students to construct a representation and make a prediction but did not require connecting the representation and prediction would not be marked as potentially engaging students in the practice of modeling. Analogous criteria exist for selected response *items* that potentially elicit evidence of student engagement in a science practice. Such criteria for developing and using models include:

Give an event, observation, or phenomenon for the student to explain or make a prediction about
Give a representation or ask the student to select a representation
Ask the student to select an explanation or select a prediction about the event, observation, or phenomenon
Ask the student to select the reasoning that links the representation to their explanation or prediction

As we used the 3D-LAP to describe exams in our data set, it became clear that, sometimes, assessment *items* would contain *subparts* built around a single focal phenomenon. In these cases, the authors consider which *subparts*, if any, were necessary to meet the criteria for a science practice and excluded decoupled *subparts* from the 3D point count. This was done to not overinflate the extent of science practices prompted for on exams. An example of this coding is provided in Figure 2.

Once we determined that an assessment *item*, or at least one of its *subparts*, met the criteria for potentially engaging students in a science practice, we considered whether this engagement required students to draw on core chemistry ideas (e.g., electrostatics and bonding interactions, energy) as framed by crosscutting concepts (e.g., cause and effect: mechanism and explanation, structure and function). Our coding described which chemistry core idea(s) and crosscutting concepts were foregrounded in each *item*/*subpart*.

The points allotted to *items*/*subparts* that had the potential to elicit (or helped contribute to eliciting) evidence of engagement in all three dimensions were summed up. The points allotted to *items*/*subparts* that only met the criteria for potentially eliciting evidence of engagement in a science practice but did not prompt for use of core ideas and/or crosscutting concepts were summed up separately. Assessment points were thus sorted into one of three categories: (1) 3D points; (2) Science Practice Only points; or (3) 0D points (i.e., points allotted to *items*/*subparts* that do not meet the 3D-LAP criteria to potentially engage students in a science practice). Patterns in code distribution were disaggregated by institution type to enable exploration of associations between institution type (e.g., R1 University, R2 University, Primarily Undergraduate Institution) and emphasis on 3D Learning. Since our data contained two mutually exclusive categorical variables and a non-normal distribution, we employed a three-by-three χ^2^ test to determine what, if any, relationship exists between institution type and 3-Dimensional Learning emphasized on assessments. Further details on all χ^2^ tests performed for this study are reported after the description of Phase 2.

### Phase 2: Development and Use of a Protocol to Describe 0D *Tasks*

Many *items* in our data set did not meet the 3D-LAP criteria for potentially eliciting evidence of 3D Learning. As such, we needed a way to describe the intellectual work emphasized by these 0D *items*. Simply saying “X% of surveyed exam points were not 3D” does not give our community much to work off of. To that end, we sought to construct a protocol with a similar form to the 3D-LAP that was capable of describing *tasks* embedded in 0D *items*/*subparts* in our data set. Protocol development was an iterative process that consisted of 4 steps:

1. The first author examined our collection of 0D assessment *items*/*subparts* and constructed analytic memos^60^ that described what inferences the responses to *tasks* embedded in an assessment *item*/*subpart* could reasonably support. For example. If we consider the *subparts* excluded from the 3D point count in Figure 2, responses to *tasks* embedded in *subparts* B, C, and E would suggest students can set up mathematical equations (*subpart* B) or use an equation to compute a numerical knowledge product (*subparts* C and E).
2. Using a constant comparative approach,^61^ the first and third authors compared and contrasted patterns in *task* types described by memos to revise, combine, and collapse them into a list of distinctly different *task* categories. Within each category, we nested *task* types that prompted for the same kind of knowledge product. For example, naming who discovered the electron, regurgitating the textbook definition of “boiling point,” and reciting procedural steps in a titration are all facts provided to students for them to recall on assessments. These seemed qualitatively distinct from questions that asked students to make predictions and/or comparisons, evaluate the validity of a claim, or provide an example of a scientific term as these potentially require students to construct their own knowledge in situ rather than recall previously constructed facts.
3. The first author generated criteria with a similar form to those used by the 3D-LAP to describe characteristics of each type of *task*. These were refined with input from the third author. For example, the criteria a *task* must meet to potentially elicit evidence of students Writing Chemical Reactions include the following:
  - Provide at least two chemical species to react;
  - Provide (or ask students to provide) the products resulting from the given reactants;
  - Provide (or ask students to provide) coefficients that follow the law of conservation of mass.
4. The first and second author applied draft protocols to 10–15% of 0D assessment *items* to evaluate whether criteria were useful in reliably describing the varied prompts that comprise our data set. Criteria for *task* types were frequently refined as a result of this process due to the complexity of our coding scheme and the variability of prompts in our data set. Additionally, all discrepancies in coding were discussed and consensus was reached. Following each round of discussion, previously coded exams were rechecked to ensure compliance with the updated protocol. After six rounds of discussion and protocol revision, the two coders were able to very consistently describe assessment *tasks* with the 0D protocol (Cohen’s Kappa = 0.895 for *task* category and 0.843 for *tasks* nested in these categories)^59^ The final version of our 0D *task* protocol is provided in Figure 3 and examples of *tasks* described by each code can be found in the .

**Figure 3:**
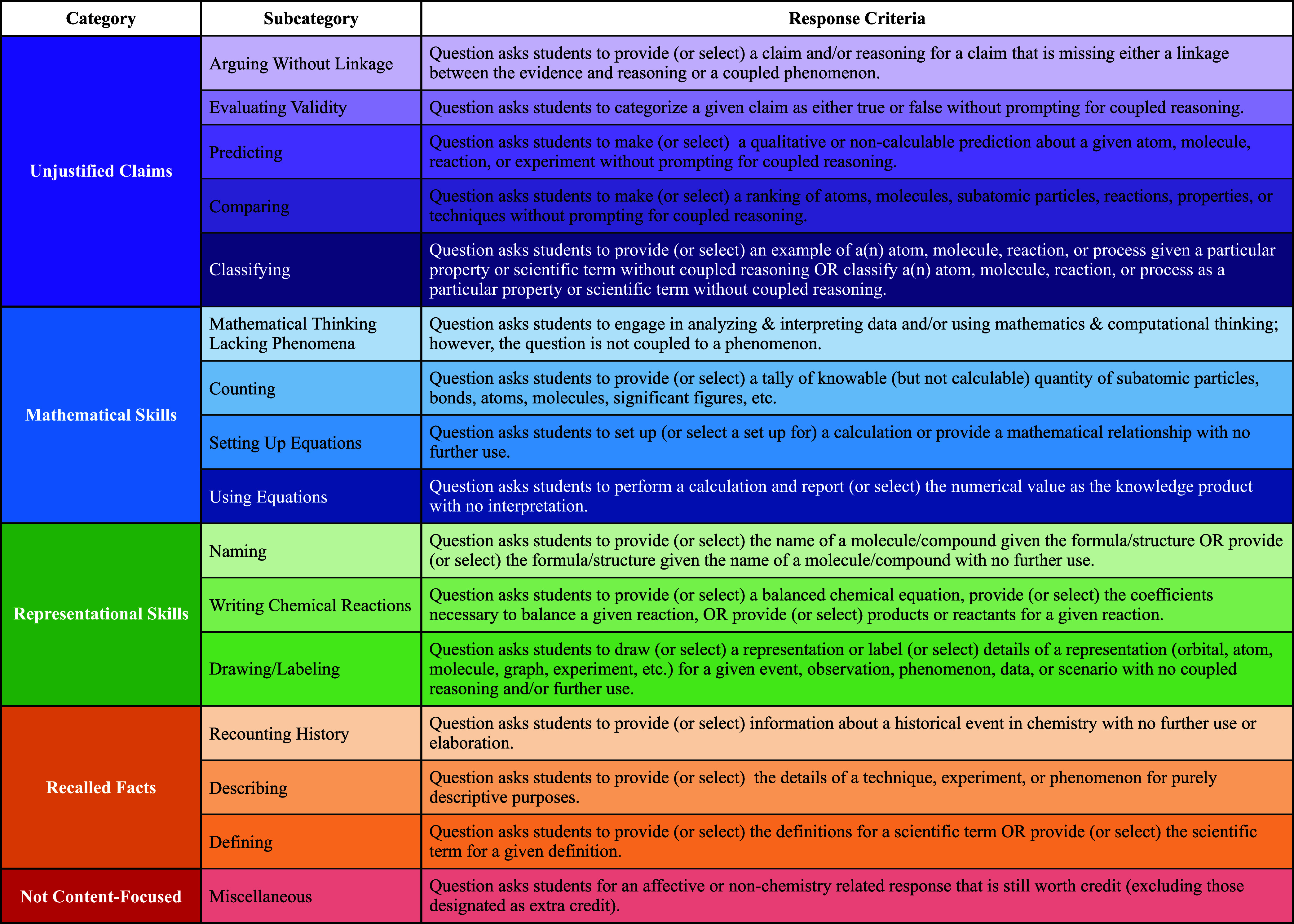
Each explicit *task* is coded allowing for multiple codes per *item*/*subpart*. This color scheme is a legend for the results presented in Figure 6.

Use of our inductively developed protocol was very similar to how we made use of the 3D-LAP (i.e., assessing what specific criteria are met and labeling the *task* accordingly) with one major difference in code reporting: we report the number of *tasks* of each type rather than the number of points dedicated to each type of *task*. This was necessary as many exams did not provide a sufficiently granular point breakdown for us to tabulate the relative weighting of each *task* type when assessment *items*/*subparts* prompted for multiple *tasks*. For example, we could not infer whether drawing the Lewis structure or labeling this structure with a molecular dipole was worth more points in 2c (Figure 1).

### Examining Associations between Institution Type and Exam Emphasis

To explore whether substantive associations existed between institution type (PUI, R1, R2) and exam emphasis, we carried out two Pearson’s χ^2^ tests using SPSS.^62^ For the first of these, we looked for an association between institution type and the distribution of exam points that were allocated to 3D *items*/*subparts*, *items*/*subparts* that emphasized only a science practice, and 0D *items*/*subparts*. For the second χ^2^ test, we analyzed associations between institution type and the distribution of 0D *task* categories. We removed the fifth category of *task*, Not Content-Focused, from this analysis because these *tasks* do not require students to use chemistry content. As such, this rare *task* type is not relevant to the overall story of ways in which exams prompt students to engage with chemistry. The output of each χ^2^ test included χ^2^ and Cramer’s *V*. Cohen’s guidelines for effect size were used to interpret Cramer’s *V*.^63^ For a contingency table containing three rows, such as the table generated by our first χ^2^ test, values of 0.212, 0.345, and 0.71 for Cramer’s *V* correspond to small, medium, and large effect sizes, respectively. The threshold of significance used for this study was *p* ≤ 0.01. A post hoc analysis of each χ^2^ test which showed a statistically significant association between variables was conducted in order to help us infer the driver(s) of that significance. Adjusted standardized residuals for each cell were calculated by SPSS. Adjusted standardized residuals with positive values indicated more counts than expected by chance while residuals with negative values indicated fewer counts than expected by chance. The magnitude of the adjusted standardized residual was compared to a critical value, which was 3 for a threshold of significance of *p* ≤ 0.01 with a table of many cells.^64,65^ Thus, cells with adjusted standardized residuals larger than 3 or smaller than −3 were considered drivers of the significant association. Importantly, significant associations are not necessarily of practical importance—association substantiveness is properly judged by effect size. Our χ^2^ analyses are intended to be descriptive of patterns in our data set and do not support instructor-level or causal claims.

## Results and Discussion

Recall that our aim with this study is to begin to sketch a national picture of what counts in general chemistry. To do so, we present and discuss the results from our two-phase data analysis. Our findings are structured around two claims we support with our empirical results. These are

- *Items* that potentially elicit evidence of 3-Dimensional Learning were extremely rare across our data set
- Types of 0D *tasks* emphasized on exams did not vary substantively across different types of institutions

We discuss each of these in turn.

### 3D *Items* Were Rare Across Our Data set

Looking across the 235 exams that comprise our data set, it was clear that *items*/*subparts* which could potentially elicit evidence of 3D Learning were extremely rare. Specifically, only 6.3% of all exam points were allotted to such 3D *items*/*subparts* (Figure 4). This means that, in most general chemistry courses we explored, one could earn a relatively high grade without ever using the big ideas of chemistry (e.g., atomic/molecular structure and properties, change and stability in chemical systems) to explain how and why chemical phenomena occur. It is important to note that emphasis on 3D Learning was unevenly distributed across our data set. Out of all 235 exams, 122 exams contained no 3D assessment *items*/*subparts* at-all (this amounts to 52% of all exams in our data set).

**Figure 4:**
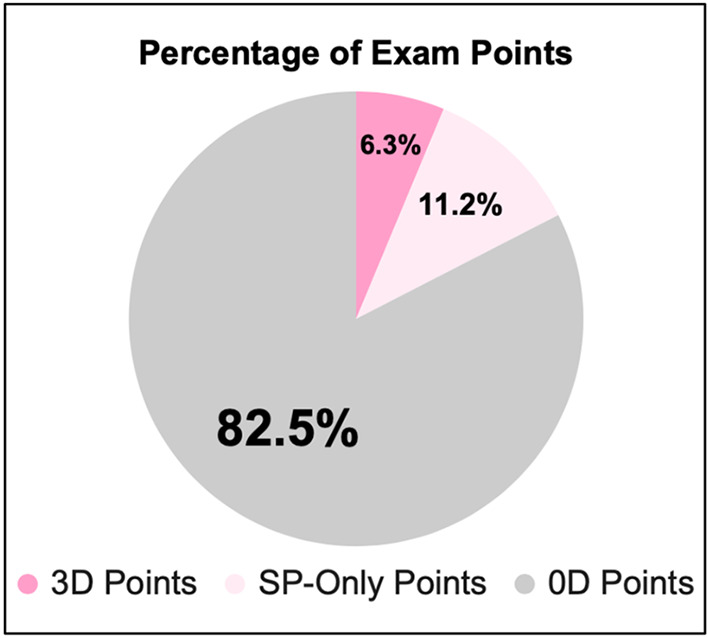
Percentage of exam points in our data set allotted to assessment items that potentially elicit—or contribute to eliciting—evidence of 3-Dimensional Learning (3D Points), evidence of engagement in a science practice without invoking core ideas (SP-Only Points), and items that do not potentially elicit such evidence (i.e., 0D Points).

When we explored the relationship between institution type and emphasis of 3D Learning on exams, we saw a statistically significant association (*p* < 0.001) with a miniscule effect size (Cramer’s *V* = 0.039). This means that, although the results of our χ^2^ test formally reject independence between institution type and exam emphasis, the observed association is of little practical importance—emphasis on 3D Learning was similarly low across all institution types. A posthoc analysis of the contingency table for this χ^2^ test (Figure 5) shows that the statistical significance of the association is primarily driven by the distribution of codes describing R2 assessment emphasis. Specifically, assessments administered in R2 contexts dedicated fewer points to 3D *items*/*subparts* than expected (i.e., the adjusted standardized residuals for the R2 3D Points cell are large and negative while the adjusted standardized residuals in the R2 0D Points cell are large and positive). These residuals describe directional deviations in our data set rather than substantive institutional patterns. The tiny effect size of the observed association indicates that emphasis on 3D Learning was remarkably consistent across institution types.

**Figure 5:**
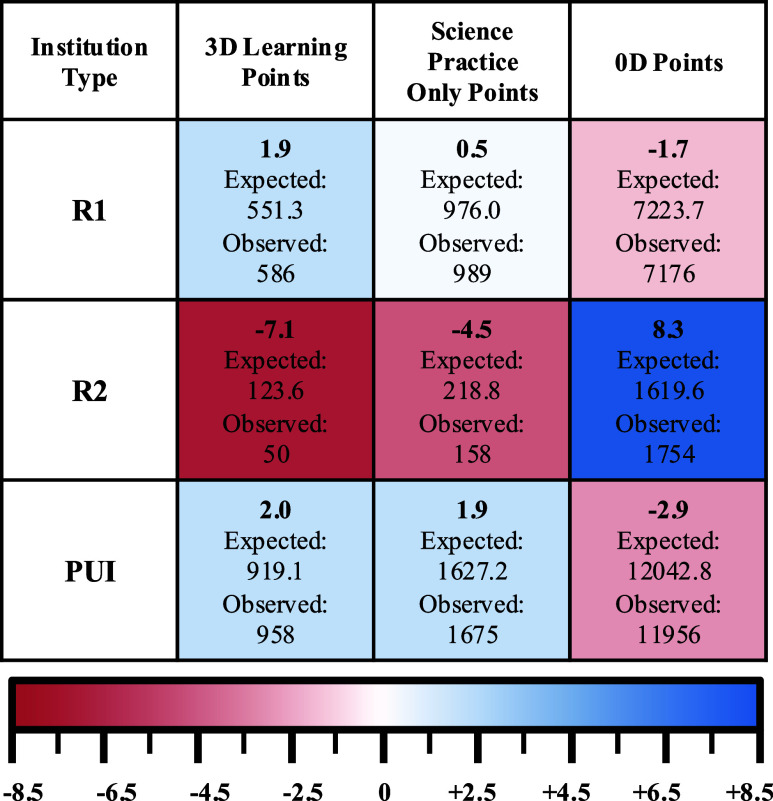
Contingency table for the χ^2^ test investigating the relationship between institution type and extent of science practices/3D Learning on general chemistry assessments. Reported with adjusted standardized residuals. *p* < 0.001. Cramer’s *V* = 0.039.

So, if 3D Learning was not emphasized much (if at all) by exams in our data set, what sort of performances were required to pass general chemistry? We turn to this next.

### Emphasis Among 0D *Tasks* Was Consistent Across All Institution Types

0D *Tasks* on exams in our data set typically fell into one of three categories: Unjustified Claims, Mathematical Skills, or Representational Skills. Within these categories, some *tasks* were emphasized more than others. For example, 69.5% of Representational Skills assessed were “Drawing/Labeling a Representation” (e.g., electron configuration, MO diagram). Likewise, within Mathematical Skills, “Using Equations” was heavily emphasized (72%). We illustrate the subcategory emphasis across our data set in Figure 6.

**Figure 6:**
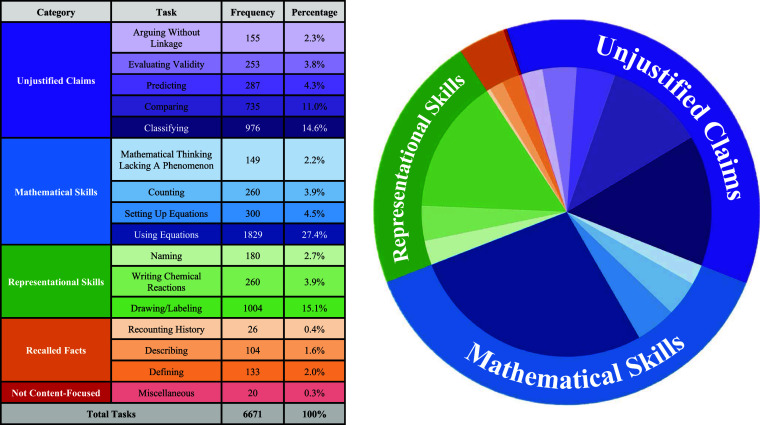
Depiction of the number of *tasks* in our data set described by each subcategory code.

Looking across three types of institutional contexts (R1, R2, PUI), we see slight variances in *task* and *task* category emphasis. For example, PUIs emphasized “Classifying” *tasks* more than R1s (16.1% and 11.5%, respectively). However, all institution types place similar emphasis on Mathematical Skills and Unjustified Claims. The most common *task* among all institution types is “Using Equations” (27.4% of all *tasks*). In fact, prompting for a numerical knowledge product with no interpretation of the meaning of this knowledge product was nearly twice as common as the second-most prompted *task* for each institution type.

When looking at the association between institution type and *task* category distribution, we see a statistically significant association (*p* < 0.001) with a very small effect size (Cramer’s *V* = 0.072). The small effect size of the association indicates that 0D *task* emphasis is consistent across institutional contexts. A posthoc analysis of the contingency table (Figure 7), shows that the statistical significance of the association is likely due to the greater emphasis of Unjustified Claim *tasks* compared to Representational Skills and Recalled Facts at R1 universities, while PUIs had the opposite emphasis in distribution of 0D *task* categories. Importantly, this residual analysis identifies cells that contribute to the χ^2^ statistic, not meaningful institutional differences.

**Figure 7:**
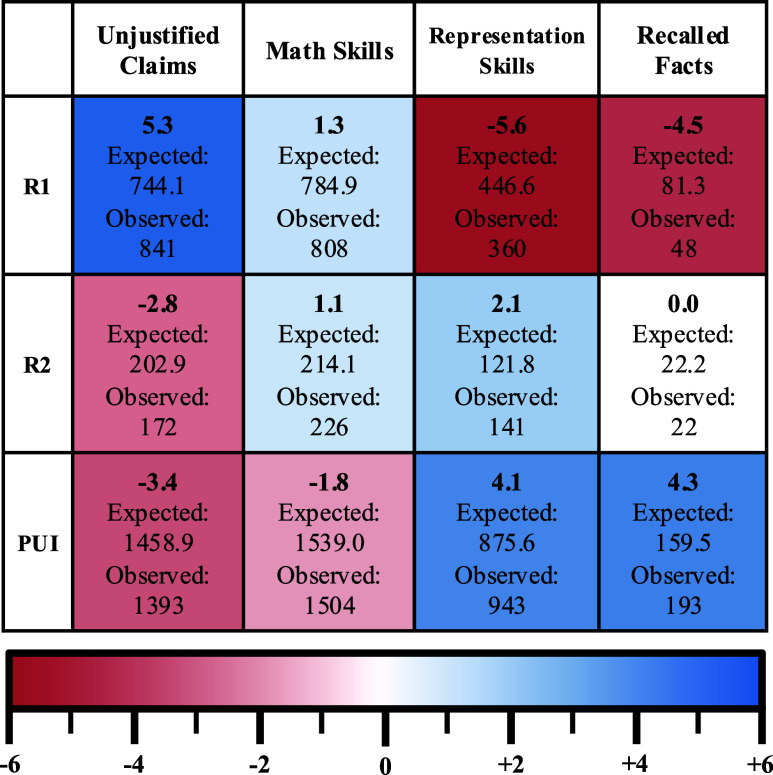
Contingency table for the χ^2^ test investigating the relationship between institution type and category of 0D *tasks* emphasized on general chemistry assessments in our data set. Reported with adjusted standardized residuals. *p* < 0.001. Cramer’s *V* = 0.072.

### What General Chemistry Exams Communicate about the Discipline

Taken together, the results of our two-phase analysis sent a clear message that school chemistry is *not* about making sense of the physical world by developing, refining, and using molecular-level models. Instead, course assessments tell students that chemistry class is a context in which one should master disconnected skills and know facts about systems that seem disconnected from the disciplinary work of chemists. Our previously reported small-scale study of three general chemistry courses was not an anomaly, but part of a larger trend of such courses emphasizing skills and facts over mechanistic explanations.^14^

When put in conversation with equity work in chemistry education,^1,6,26^ our findings suggest a reason why general chemistry disproportionately limits the futures of minoritized students (e.g., Black, Latine, and first-generation):^1,66^ success in general chemistry requires executing math skills on exams. Since successful performance of these skills is a proxy for access to precollege math preparation,^6,17^ general chemistry sorts students based on how well-resourced their high school math program was. Note that we are not advocating for the removal of math from general chemistry. As mentioned earlier, there are many chemical phenomena that require generating or interpreting mathematical representations to understand. Instead, we are connecting this and prior empirical work to say that the general chemistry exams we characterized placed substantial emphasis on *task* types that have been observed to discriminate on the basis of access to precollege math preparation.^6^ We encourage our community to carefully consider when and how mathematical skills should be used to support students’ sensemaking of chemical phenomena. We also urge our community to investigate approaches to math that are more equitable than the status quo.

To be clear, we do not claim that emphasizing 3D Learning on assessments will invariably lead to equity. Indeed, one might argue that valorizing a vision of professional science as *The Framework* does runs counter to equity discourses that seek to shift what and whose knowledge is considered scientific.^67^ Readers interested in advancing equity are encouraged to seek out frameworks purpose-built to accomplish this goal. For example, Scanlon et al.^68^ has published work investigating the accessibility and equity of a selection of curricular reforms using a framework based on Universal Design for Learning (UDL).^69^ Equity-centered frameworks, in addition to 3D Learning can help us envision and enact more equitable and meaningful assessment approaches.

### Implications for Teaching

Given the findings reported here, one might ask how our community might improve matters. Specifically, how can we work toward courses that potentially engage our students in constructing, refining, and communicating molecular-level causal accounts for phenomena? One productive step, we claim, is to shift the emphasis of high- and low-stakes assessments in our courses. Fortunately, it can be relatively straightforward to transform a 0D *item* into a 3D *item*.^70^ For example, suppose we typically give 0D *items* such as those shown in Figure 8 (left pane). Responses to *subparts* of this *item* can tell us something about student facility with labeling skills (e.g., drawing lone pairs), and their ability to write normative claims about solubility. However, credit-earning responses to these *subparts* provide little to no evidence as to **why** students wrote the claims they did. Students may have written, “oxycodone is highly insoluble in water ,” because they guessed or because they considered the energetics of oxycodone dissolution in water. To elicit stronger evidence that students were grounding their claims in chemistry big ideas, we would need to elaborate on the framework provided by the 0D *subparts*. We would need to ask students to—explicitly in writing—connect their drawing of the IMFs that occur between oxycodone and water to their claims about solubility. We could then build from this to ask students to consider how the solubility of oxycodone might be affected by dropping it in stomach acid. Here too, to elicit evidence that students’ reasoning was connected to big ideas, we would need to explicitly prompt for these connections. The right pane of Figure 8 provides an example of what a more elaborated 3D prompt might look like for this phenomenon context. Importantly, while this 3D *item* has the potential to elicit evidence that students are using big ideas to explain phenomena, we would need to give this *item* to students to know whether it was successful in eliciting such evidence.

**Figure 8:**
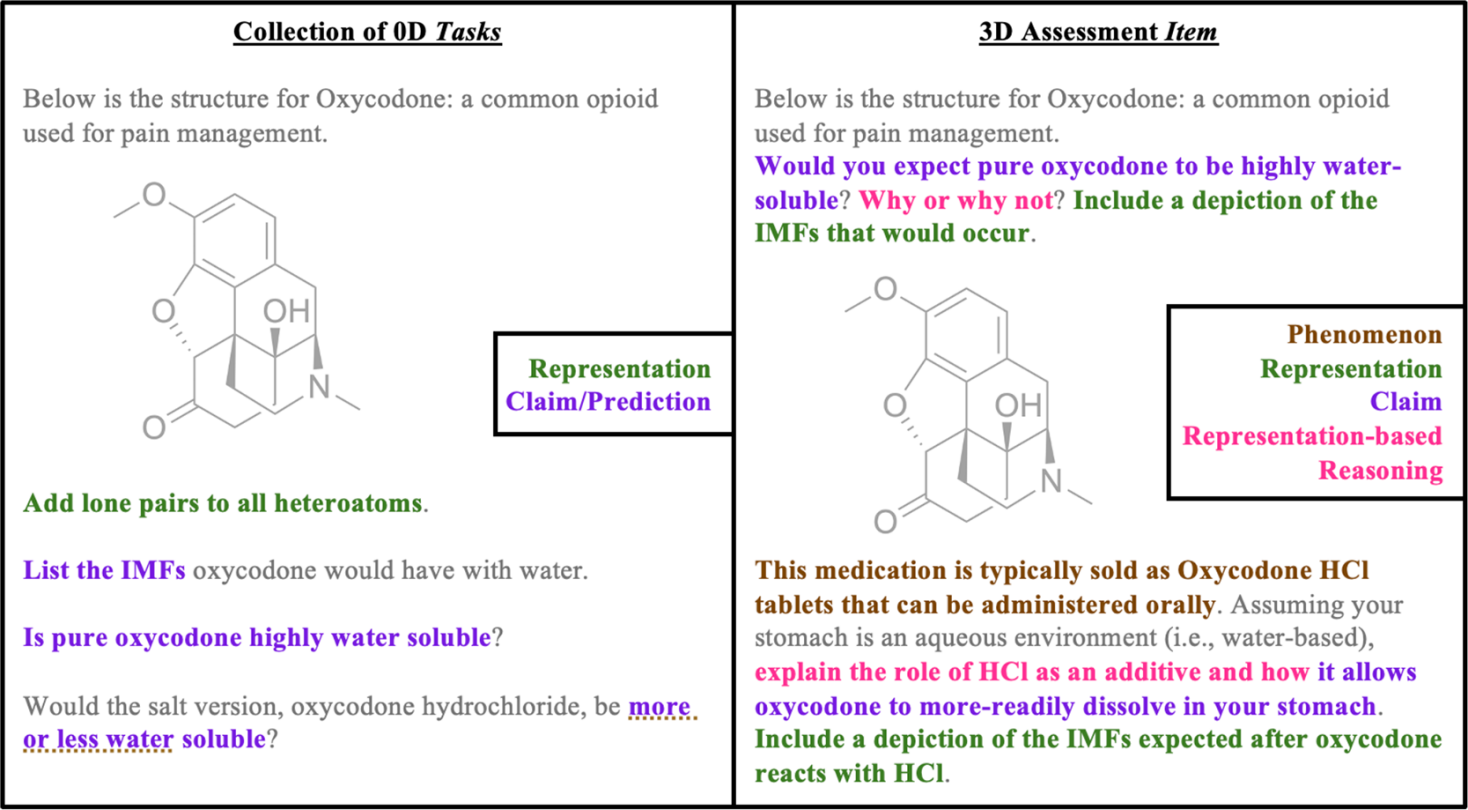
Sample assessment *item* that incorporates intermolecular forces but does not meet 3D-LAP criteria (left) and assessment *item* that incorporates intermolecular forces while meeting 3D-LAP criteria (right).

To support successful engagement with 3D *items* (e.g., Figure 8, right pane), we must build and enact general chemistry courses that effectively support and scaffold 3D Learning. There are several examples in our field that provide a template for what such support might look like. Chemistry, Life, the Universe, and Everything (or CLUE)^71^ builds the course narrative around using big ideas in the discipline to explain and model increasingly complex phenomena. Chemical Thinking^72^ similarly organizes class activity around fundamental questions that require students to connect big ideas to how and why phenomena occur. If adopting and adapting a new curriculum is not feasible, instructors may also slowly evolve a traditional course to increasingly emphasize 3D *items* on exams. Doing so requires consistent reflection on (mis)­alignment between exams and support materials meant to prepare students for those exams. We have followed this slow-and-steady approach to developing 3-Dimensional organic chemistry courses at UW–Madison.^49,50,73^ The pace and magnitude of change that is manageable will of course vary depending on the time and resources available to an instructor or instructional team. We therefore encourage instructors to start with “low hanging fruit” (i.e., *tasks* that might be made 3D by simply prompting for reasoning or centering attention on an observable phenomenon) and building out from there. Relatedly, if one is concerned about the time and effort required to grade elaborate, constructed response problems (e.g., Figure 8), we encourage adapting these into a selected response format. A prompt in which students select relevant representations, predictions/explanations, and reasoning to model a phenomenon is an improvement on a prompt in which students simply draw a decontextualized picture.

Importantly, we should also be realistic about what shifting toward more 3D assessments can accomplish. Empirical work carried out by our group suggests that students in a 3D class sometimes see topics and phenomena as interconnected and explainable by using a relatively small set of core ideas.^8^ For example, Sara (pseudonym), a chemistry major in a transformed 3D organic course noted that (emphasis ours):

I think that **most of the science classes I’ve taken so far have been more of lists of topics and you have to kind of review all of them specifically and they do not really add up to something bigger the way that this chemistry class does**. And even when I was in [general chemistry II] last semester, we learned acids/bases and kinetics and reactions rates and all those separate concepts, but **we did not really put them together into something bigger the way that we did in this class**.

This is certainly an improvement relative to experiencing chemistry class as a disconnected bundle of facts and skills. However, despite experiencing the class as more coherent, Sara still saw her role as constructing explanations, models, etc. that looked like what the instructor wrote on the answer key.^8^ More ambitious reimaginings of chemistry teaching will be needed to move us toward supporting ways of thinking that are, in fact, useful in personal or professional life.^28^

### Implications for Research

This study provides a coarse-grained look at what is emphasized and rewarded on a large number of general chemistry exams given in diverse institutional contexts. Our findings suggest that assessment reforms championed by the chemistry education community have had little impact on what is assessed in introductory chemistry. If we are committed to shifting this status quo, then empirical studies situated in chemistry courses should attend to—and work to shift—assessment emphasis. Statements about “increased student learning” or “increased student success” should be accompanied by descriptions of what was learned and how we know. It would also be worthwhile for members of our community to explore ways in which general chemistry assessment emphasis is stabilized by institutional policies and practices. Such work might surface taken-for-granted assumptions that constrain how and what we can imagine assessing.

### Limitations

Like all empirical studies, this work is limited in a variety of ways. First, we solicited and obtained exams from institutions located in the United States and cannot comment on what non-US contexts assess. Second, although our exam data set is large, it is not comprehensive. We cannot make claims about what is emphasized and rewarded in courses that were not represented in our data set. Third, our approach to coding was reductive—we described what competencies each *item*/*subpart* or *task* had the potential to elicit evidence of. Codes were mutually exclusive—one *task* could only be assigned a single code. This approach let us parse an extremely complex and varied data set in a way that (we hope) is clear to the reader. Importantly, our codes should be read as the authors’ sense of what inferences would be supportable using data from prompt responses. There are certainly other inferences one could make from responses to a *task* that our coding did not capture.

## Conclusions

With this study, we set out to provide a nuanced look at the nature of performances emphasized and rewarded in general chemistry across the United States. While our picture is incomplete, we observed that the vast majority of exams in our data set emphasized little to no 3D Learning. This means that students can (and do) achieve high marks in many general chemistry classes without ever using powerful explanatory ideas (e.g., energy, electrostatics and bonding interactions) to construct causal accounts for chemical phenomena. Instead, success in gatekeeper chemistry courses principally comes about by using mathematical skills, constructing unjustified claims, and drawing/labeling specialist pictures. This assessment emphasis tells our students that chemistry is about performing a jumble of decontextualized skills. We suspect this is at odds with how most chemists understand their discipline.

In addition to misrepresenting the discipline of chemistry, our field’s overemphasis on a grab-bag of skills has material consequences for students’ possible futures. Since general chemistry is a prerequisite for many STEM and STEM-adjacent majors and careers, we are, in effect, saying, “you can only be a physician, dentist, marine biologist, etc. if you draw arcane pictures and perform obtuse math problems.” This is unreasonable, especially in light of the documented role general chemistry plays in preventing Black and Brown students from pursuing careers in STEM.^1,6,26^ One can be a fantastic physician or dentist without quickly generating the electron configuration for ytterbium.

Our findings should not be read as suggesting ill intent on the part of instructors who administered the exams in our data set. In our experience, most chemistry instructors are working hard to support what they see as useful chemistry learning. Unfortunately, the foundations of dominant conceptualizations of “useful chemistry learning” in our field are rarely surfaced or questioned. Instead, it is often assumed that the skills and facts deemed important by professional chemists in the 1960s are indeed critical for everyone from future chemists to future dentists. This assumption runs counter to decades of research in Science & Technology Studies (STS), which demonstrates that knowledge of scientific content is seldom useful in tackling the practical problems of real people.^74–76^ It would be worth our community thinking carefully about for whom the content in our courses is useful and for what purpose. As we do so, we should dialogue with our students, their communities, and colleagues in fields such as STS.

Our field has produced curricula^71,72^ and approaches to assessment writing^70,77^ that foregrounded using energy and bonding to make sense of phenomena. It is possible, though nontrivial, to focus student attention on making sense of the natural world in terms of molecular interactions. Others in the larger science education community have called for an even more fundamental rethink of what science education should entail.^78–82^ These colleagues encourage us to move beyond attending to what the chemical sciences can do and toward interrogating who benefits and who is harmed by analyzing and manipulating matter in certain ways. We hope our findings serve as a call to engage with colleagues in the broader science education community to envision chemistry assessments that are both equitable and meaningful.

## Supplementary Material





## Notes

0D task coding protocol (PDF)
Distributions by institution type (PDF)

## References

[1] Harris R. B., Mack M. R., Bryant J., Theobald E. J., Freeman S. (2020). Reducing Achievement
Gaps in Undergraduate General Chemistry Could Lift Underrepresented
Students into a “Hyperpersistent Zone”. Sci. Adv..

[2] Matz R. L., Koester B. P., Fiorini S., Grom G., Shepard L., Stangor C. G., Weiner B., McKay T. A. (2017). Patterns of Gendered
Performance Differences in Large Introductory Courses at Five Research
Universities. AERA Open.

[3] Alexander C., Chen E., Grumbach K. (2009). How Leaky
Is the Health Career Pipeline?
Minority Student Achievement in College Gateway Courses. Acad. Med..

[4] Gibbons R. E., Reed J. J., Srinivasan S., Murphy K. L., Raker J. R. (2022). Assessment
Tools in Context: Results from a National Survey of Postsecondary
Chemistry Faculty. J. Chem. Educ..

[5] Ralph V. R., Scharlott L. J., Schwarz C. E., Becker N. M., Stowe R. L. (2022). Beyond
Instructional Practices: Characterizing Learning Environments That
Support Students in Explaining Chemical Phenomena. J. Res. Sci. Teach..

[6] Ralph V. R., Scharlott L. J., Schafer A. G. L., Deshaye M. Y., Becker N. M., Stowe R. L. (2022). Advancing
Equity in STEM: The Impact Assessment Design
Has on Who Succeeds in Undergraduate Introductory Chemistry. JACS Au.

[7] Snyder, B. R. The Hidden Curriculum; MIT Press: Cambridge, MA, 1973.

[8] Schwarz C. E., DeGlopper K. S., Greco N. C., Russ R. S., Stowe R. L. (2025). Modeling
Student Negotiation of Assessment-Related Epistemological Messages
in a College Science Course. Sci. Educ..

[9] Momsen J., Offerdahl E., Kryjevskaia M., Montplaisir L., Anderson E., Grosz N. (2013). Using Assessments
to Investigate
and Compare the Nature of Learning in Undergraduate Science Courses. CBE—Life Sci. Educ..

[10] Scouller K. (1998). The Influence
of Assessment Method on Students’ Learning Approaches: Multiple
Choice Question Examination versus Assignment Essay. High. Educ..

[11] Scouller K. M., Prosser M. (1994). Students’ Experiences in Studying for Multiple
Choice Question Examinations. Stud. High. Educ..

[12] Entwistle N. J. (1991). Approaches
to Learning and Perceptions of the Learning Environment: Introduction
to the Special Issue. High. Educ..

[13] Crooks T. J. (1988). Impact
of Classroom Evaluation Practices on Students. Rev. Educ. Res..

[14] Stowe R. L., Scharlott L. J., Ralph V. R., Becker N. M., Cooper M. M. (2021). You Are
What You Assess: The Case for Emphasizing Chemistry on Chemistry Assessments. J. Chem. Educ..

[15] Sanabria-Ríos D., Bretz S. L. (2010). Investigating
the Relationship between Faculty Cognitive
Expectations about Learning Chemistry and the Construction of Exam
Questions. Chem. Educ Res. Pr..

[16] James N. M., LaDue N. D. (2021). Pedagogical Reform
in an Introductory Chemistry Course
and the Importance of Curricular Alignment. J. Chem. Educ..

[17] Rosa V., States N. E., Corrales A., Nguyen Y., Atkinson M. B. (2022). Relevance
and Equity: Should Stoichiometry Be the Foundation of Introductory
Chemistry Courses?. Chem. Educ. Res. Pract..

[18] Philosophy of Chemistry: Growth of a New Discipline, Scerri, E. R. ; Mclntyre, L. , Eds.; Boston Studies in the Philosophy and History of Science; Springer: Dordrecht, The Netherlands, 2015; 10.1007/978-94-017-9364-3.

[19] Martin D. B. (2009). Researching
Race in Mathematics Education. Teach. Coll.
Rec. Voice Scholarsh. Educ..

[20] Diversity in Mathematics Education Center for Learning and Teaching . Culture, Race, Power, and Mathematics Education. In Second handbook of research on mathematics teaching and learning: a project of the National Council of Teachers of Mathematics; Lester, F. K. , Ed.; Information Age: Charlotte, NC, 2007; pp 405–433. 10.1108/978-1-28141-200-3.

[21] Russ R. S. (2018). Characterizing
Teacher Attention to Student Thinking: A Role for Epistemological
Messages: TEACHER ATTENTION TO STUDENT THINKING. J. Res. Sci. Teach..

[22] Zammit K. M., Connor M. C., Raker J. R. (2026). Instructional and Assessment Practices
Used in Chemistry Courses at Two-Year Institutions: Results of a National
Survey. J. Chem. Educ..

[23] Witherspoon E. B., Vincent-Ruz P., Schunn C. D. (2019). When Making the Grade Isn’t
Enough: The Gendered Nature of Premed Science Course Attrition. Educ. Res..

[24] King N. S., Pringle R. M. (2019). Black Girls Speak STEM: Counterstories
of Informal
and Formal Learning Experiences. J. Res. Sci.
Teach..

[25] Vincent-Ruz P., Binning K., Schunn C. D., Grabowski J. (2018). The Effect
of Math SAT on Women’s Chemistry Competency Beliefs. Chem. Educ. Res. Pract..

[26] Rosa V., Lewis S. E. (2018). Chemistry Topics
Posing Incommensurate Difficulty to
Students with Low Math Aptitude Scores. Chem.
Educ. Res. Pract..

[27] Robinson K. A., Perez T., Carmel J. H., Linnenbrink-Garcia L. (2019). Science Identity
Development Trajectories in a Gateway College Chemistry Course: Predictors
and Relations to Achievement and STEM Pursuit. Contemp. Educ. Psychol..

[28] Stowe R. L. (2026). Getting
Serious about Useful Chemistry Learning: A Case for Attending to Epistemological
Messaging. J. Chem. Educ..

[29] Ashford T. A. (1955). The Examinations
Program of the Division of Chemical Education: A Committee Report
for the Period 1947–54. J. Chem. Educ..

[30] Nakhleh M. B. (1993). Are Our
Students Conceptual Thinkers or Algorithmic Problem Solvers? Identifying
Conceptual Students in General Chemistry. J.
Chem. Educ..

[31] Stamovlasis D., Tsaparlis G., Kamilatos C., Papaoikonomou D., Zarotiadou E. (2005). Conceptual
Understanding versus Algorithmic Problem
Solving: Further Evidence from a National Chemistry Examination. Chem. Educ Res. Pr..

[32] Zoller U. (1993). Are Lecture
and Learning Compatible? Maybe for LOCS: Unlikely for HOCS. J. Chem. Educ..

[33] Holme T. A., Luxford C. J., Brandriet A. (2015). Defining Conceptual Understanding
in General Chemistry. J. Chem. Educ..

[34] Stowe R. L., Cooper M. M. (2017). Practicing What We Preach: Assessing “Critical
Thinking” in Organic Chemistry. J. Chem.
Educ..

[35] A Framework for K–12 Science Education: Practices, Crosscutting Concepts, and Core Ideas; National Academies Press: Washington, D.C., 2012; p 13165. 10.17226/13165.

[36] Reed J. J., Brandriet A. R., Holme T. A. (2017). Analyzing the Role of Science Practices
in ACS Exam Items. J. Chem. Educ..

[37] Bowen R. S. (2025). On a More
Expansive Vision for the Scientific Practices in Undergraduate Chemistry
Education. J. Chem. Educ..

[38] Bapu
Ramesh V., Seery M. K., Cole R. (2026). Assessing Science Practices
in Undergraduate Chemistry Laboratories: Why We Need to Do Better—and
How We Should. J. Chem. Educ..

[39] Pazicni S., Wink D. J., Donovan A., Conrad J. A., Darr J. P., Morgan Theall R. A., Richter-Egger D. L., Villalta-Cerdas A., Walker D. R. (2021). The American Chemical
Society General Chemistry Performance
Expectations Project: From Task Force to Distributed Process for Implementing
Multidimensional Learning. J. Chem. Educ..

[40] Pazicni S., Flynn A. B. (2019). Systems Thinking in Chemistry Education: Theoretical
Challenges and Opportunities. J. Chem. Educ..

[41] Carmel J. H., Herrington D. G., Posey L. A., Ward J. S., Pollock A. M., Cooper M. M. (2019). Helping Students to “Do Science”: Characterizing
Scientific Practices in General Chemistry Laboratory Curricula. J. Chem. Educ..

[42] Cooper M. M., Caballero M. D., Ebert-May D., Fata-Hartley C. L., Jardeleza S. E., Krajcik J. S., Laverty J. T., Matz R. L., Posey L. A., Underwood S. M. (2015). Challenge Faculty to Transform STEM
Learning. Science.

[43] Latour, B. ; Woolgar, S. ; Salk, J. Laboratory Life: The Construction of Scientific Facts; Princeton University Press: Princeton, NJ, 2013. 10.1515/9781400820412.

[44] Pickering, A. The Mangle of Practice: Time, Agency, and Science; University of Chicago Press: Chicago, 2010.

[45] Laverty J.
T., Underwood S. M., Matz R. L., Posey L. A., Carmel J. H., Caballero M. D., Fata-Hartley C. L., Ebert-May D., Jardeleza S. E., Cooper M. M. (2016). Characterizing College Science Assessments:
The Three-Dimensional Learning Assessment Protocol. PLoS One.

[46] Lutter J. C., Hale L. V. A., Shultz G. V. (2019). Unpacking
Graduate Students’
Knowledge for Teaching Solution Chemistry Concepts. Chem. Educ. Res. Pract..

[47] Cooper M. M., Caballero M. D., Carmel J. H., Duffy E. M., Ebert-May D., Fata-Hartley C. L., Herrington D. G., Laverty J. T., Nelson P. C., Posey L. A., Stoltzfus J. R., Stowe R. L., Sweeder R. D., Tessmer S., Underwood S. M. (2024). Beyond Active Learning: Using 3-Dimensional
Learning to Create Scientifically Authentic, Student-Centered Classrooms. PLoS One.

[48] Matz R. L., Fata-Hartley C. L., Posey L. A., Laverty J. T., Underwood S. M., Carmel J. H., Herrington D. G., Stowe R. L., Caballero M. D., Ebert-May D., Cooper M. M. (2018). Evaluating the Extent of a Large-Scale
Transformation in Gateway Science Courses. Sci.
Adv..

[49] Schwarz C. E., DeGlopper K. S., Esselman B. J., Stowe R. L. (2024). Tweaking Instructional
Practices Was Not the Answer: How Increasing the Interactivity of
a Model-Centered Organic Chemistry Course Affected Student Outcomes. J. Chem. Educ..

[50] Esselman B. J., Hill N. J., DeGlopper K. S., Ellison A. J., Stowe R. L., Schwarz C. E., Ellias N. J. (2023). Authenticity-Driven
Design of a High-Enrollment
Organic Laboratory Course. J. Chem. Educ..

[51] Talanquer V. (2018). Importance
of Understanding Fundamental Chemical Mechanisms. J. Chem. Educ..

[52] Cooper M. M., Posey L. A., Underwood S. M. (2017). Core Ideas
and Topics: Building Up
or Drilling Down?. J. Chem. Educ..

[53] Zoller U. (2002). Algorithmic,
LOCS and HOCS (Chemistry) Exam Questions: Performance and Attitudes
of College Students. Int. J. Sci. Educ..

[54] Momsen J. L., Long T. M., Wyse S. A., Ebert-May D. (2010). Just the Facts?
Introductory Undergraduate Biology Courses Focus on Low-Level Cognitive
Skills. CBE—Life Sci. Educ..

[55] Bloom, B. S. Taxonomy of Educational Objectives: The Classification of Educational Goals, First; Longmans, Green: New York, 1956.

[56] U.S. News & World Report . Best National Universities Rankings. https://www.usnews.com/best-colleges/rankings/national-universities?myCollege=national-universities&_sort=myCollege&_sortDirection=asc(accessed 2021-04-13).

[57] U.S. News & World Report . Best National Liberal Arts Colleges Rankings. Best National Liberal Arts Colleges Rankings. https://www.usnews.com/best-colleges/rankings/national-liberal-arts-colleges?myCollege=national-liberal-arts-colleges&_sort=myCollege&_sortDirection=asc(accessed 2021-04-13).

[58] National Research Council . Knowing What Students Know: The Science andDesign of Educational Assessment; National Academies Press: Washington, D.C., 2001; p 10019. 10.17226/10019.

[59] Watts F. M., Finkenstaedt-Quinn S. A. (2021). The Current
State of Methods for
Establishing Reliability in Qualitative Chemistry Education Research
Articles. Chem. Educ. Res. Pract..

[60] Marshall, C. ; Rossman, G. B. ; Blanco, G. L. Designing Qualitative Research, 7th ed., SAGE: Los Angeles, 2022 (International Student Edition).

[61] Glaser, B. G. ; Strauss, A. L. The Discovery of Grounded Theory: Strategies for Qualitative Research; Aldine: Chicago, 1967.

[62] IBM . IBM SPSS Statistics 2025.

[63] Cohen, J. Statistical Power Analysis for the Behavioral Sciences, 2nd ed.; Erlbaum Associates: Hillsdale, NJ, 1988. 10.4324/9780203771587.

[64] MacDonald P. L., Gardner R. C. (2000). Type I Error Rate Comparisons of Post Hoc Procedures
for I j Chi-Square Tables. Educ. Psychol. Meas..

[65] Agresti, A. An Introduction to Categorical Data Analysis, 3rd ed.; Wiley Series in Probability and Statistics; Wiley: Hoboken, NJ, 2019.

[66] Castle S. D., Byrd W. C., Koester B. P., Pearson M. I., Bonem E., Caporale N., Cwik S., Denaro K., Fiorini S., Li Y., Mead C., Rypkema H., Sweeder R. D., Valdivia
Medinaceli M. B., Whitcomb K. M., Brownell S. E., Levesque-Bristol C., Molinaro M., Singh C., McKay T. A., Matz R. L. (2024). Systemic
Advantage Has a Meaningful Relationship with Grade Outcomes in Students’
Early STEM Courses at Six Research Universities. Int. J. STEM Educ..

[67] Philip T. M., Azevedo F. S. (2017). Everyday Science Learning and Equity:
Mapping the Contested
Terrain. Sci. Educ..

[68] Scanlon E., Legron-Rodriguez T., Schreffler J., Ibadlit E., Vasquez E., Chini J. J. (2018). Postsecondary
Chemistry Curricula and Universal Design
for Learning: Planning for Variations in Learners’ Abilities,
Needs, and Interests. Chem. Educ. Res. Pract..

[69] Meyer, A. ; Rose, D. H. Universal Design for Learning: Principles, Framework, and Practice; Gordon, D. T. , Ed.; CAST Professional: Lynnfield, MA, 2024. (Updated ed. of Anne Meyer and David H. Rose’s foundational text.)

[70] Underwood S. M., Posey L. A., Herrington D. G., Carmel J. H., Cooper M. M. (2018). Adapting
Assessment Tasks To Support Three-Dimensional Learning. J. Chem. Educ..

[71] Cooper M., Klymkowsky M. (2013). Chemistry,
Life, the Universe, and Everything: A New
Approach to General Chemistry, and a Model for Curriculum Reform. J. Chem. Educ..

[72] Sevian H., Talanquer V. (2014). Rethinking
Chemistry: A Learning Progression on Chemical
Thinking. Chem. Educ Res. Pr..

[73] Esselman B. J., DeGlopper K. S., Gavin S. J., Stowe R. L., Anzovino M. E., Hill N. J. (2025). Spectroscopic
Unknown Puzzles from Real Data—A
More Authentic Pedagogical Approach with Epistemological Implications. J. Chem. Educ..

[74] Feinstein N. (2011). Salvaging
Science Literacy. Sci. Educ..

[75] Feinstein N. W., Waddington D. I. (2020). Individual
Truth Judgments or Purposeful, Collective
Sensemaking? Rethinking Science Education’s Response to the
Post-Truth Era. Educ. Psychol..

[76] Layton, D. ; Jenkins, E. ; Macgill, S. ; Davey, A. Inarticulate Science? Perspectives on the Public Understanding of Science and Some Implications for Science Education; Studies in Education: Driffield, U.K., 1993.

[77] Talanquer V. (2019). Some Insights
into Assessing Chemical Systems Thinking. J.
Chem. Educ..

[78] Morales-Doyle D. (2017). Justice-centered
Science Pedagogy: A Catalyst for Academic Achievement and Social Transformation. Sci. Educ..

[79] Bang M., Warren B., Rosebery A. S., Medin D. (2013). Desettling Expectations
in Science Education. Hum. Dev..

[80] Bang M., Medin D. (2010). Cultural Processes
in Science Education: Supporting the Navigation
of Multiple Epistemologies: Improving Science Learning for Indigenous
Students. Sci. Educ..

[81] Bang M., Vossoughi S. (2016). Participatory
Design Research and Educational Justice:
Studying Learning and Relations Within Social Change Making. Cogn. Instr..

[82] Morales-Doyle D. (2023). Putting Science
Education in Its Place: The Science Question in Social Justice Education. Cult. Stud. Sci. Educ..

