# Supplementary material for: What Counts in General Chemistry? An Exploratory Look at What Was Emphasized and Rewarded on General Chemistry Exams Across the United States
Source: J Chem Educ. 2026 Jul 5;103(8):4086–98. doi: 10.1021/acs.jchemed.6c00309 (PMC13472132; doi:10.1021/acs.jchemed.6c00309)
Supplement: Supplementary file 1 [file ed6c00309_si_001.pdf]

Supporting Information

What Counts in General Chemistry? An Exploratory Look at What Was Emphasized and Rewarded on General Chemistry Exams Across the United States

Nicole C. Greco, Niall J. Ellias, and Ryan L. Stowe*

*Correspondence to rstowe@chem.wisc.edu

Department of Chemistry, University of Wisconsin – Madison, 1101 University Avenue, Madison, Wisconsin 53706-1322, United States

0D Detailed Protocol Notes

General Coding Notes 2
0D Task Protocol 4
Category 1: Unjustified Claims 5
Code 11: Predicting 5
Code 12: Comparing 6
Code 13: Evaluating Validity 7
Code 14: Classifying 8
Code 15: Arguing Without Linkage 8
Category 2: Mathematical Skills 9
Code 21: Setting Up Equations 9
Code 22: Using Equations 9
Code 23: Counting 10
Code 24: Mathematical Thinking Lacking Phenomena 10
Category 3: Representational Skills 11
Code 31: Drawing/Labeling 11
Code 32: Naming 12
Code 33: Writing Chemical Reactions 12
Category 4: Recalled Facts 13
Code 41: Recounting History 13
Code 42: Defining 13
Code 43: Describing 14
Category 5: Not Content-Focused 15
Code 51: Miscellaneous 15

General Coding Notes

- We are strict on the “interpretation” criteria for analyzing and interpreting data / using mathematics and computational thinking.
    - Asking to just label a point on a graph is NOT interpreting that graph. There needs to be an extrapolated conclusion from the data.
- We are defining “phenomena” as:
    - More than just a molecule
        - Ex: Q1 provides two molecules and asks the student to identify the atom with the largest radius and provide a coupled scientific principle and reasoning for the elicited claim. The existence of something does not qualify as “a given phenomenon, event, or observation” and would therefore not meet the criteria for a scientific practice. These questions are then coded as code 15: arguing without linkage, because it is a solid explanation without linkage to a phenomenon.
    - Not hypothetical things
        - Ex: Q1 provides rate data for a generic reaction: A + B $\leftrightharpoons$ C and asks students to use the data to determine the rate law for the reaction. This “fake” data would not be enough to consider the prompt as the scientific practice “analyzing and interpreting data” OR “using mathematics and computational thinking.” Therefore, the question would be coded as code 24: mathematical thinking lacking phenomena.
    - Not a made-man theory
        - Ex: Q1 asks students to draw three resonance structures for SCN and predict which Lewis structure would be the most stable and justify their choice with ideas of bonding. While this could be considered a representation used to make and justify a prediction, the question does not provide a phenomenon, event, or observation and therefore cannot be considered the scientific practice of “developing and using models.” This question would be coded as code 15: arguing without linkage as it is missing a link to an observable.
- We do NOT couple non-3D questions the way we coupled questions for scientific practices and 3D assessment items.
    - If questions are layered, we assume the previous information had already been provided.
        - Ex: Q1 asks students to draw Lewis structures for two different molecules (31: drawing). Q2 then asks which compound has the higher boiling point based on the structures and to select a justification, however the choices do NOT meet the criteria for the scientific practice for developing and using models (15: arguing without linkage). We can assume the structures were already provided for Q2 and not coupled the questions together as both contributing to code 15: arguing without linkage.
    - This does not apply for questions grounded in the same focal chemical entity.

        - Ex: Q1 asks students to draw the Lewis structure for a particular molecule (31: drawing) and identify the geometry (14: classifying) of the same molecule. Because the assessment prompt does not provide the structure for the “identify” action item, it cannot be coded as a “draw/label” task; it would be coded as a “classifying” task in any other independent situation without a provided structure.
- Explicit prompts need to involve different phenomena, atoms, molecules, etc. to qualify as multiple of the same code (i.e., count as discrete tasks). Different codes can apply to the same phenomena, atoms, molecules, etc.
    - This is to mitigate instances where prompts specify a particular number of things versus a vague prompt for potentially multiple things.
        - Ex: Q1 asks students to draw two additional resonance structures for the carbonate structure provided. This is only one 31 code (drawing) and not two 31 codes.
        - Ex: Q1 provides the skeletal structure for a certain molecule and asks students to provide the atom hybridization for several DIFFERENT atoms indicated in the structure. Each hybridization prompted for would be its own code 31: labeling as they are different atoms and it is an explicit choice to assess this skill in multiple different bonding scenarios.

0D Task Protocol

Category	Subcategory	Response Criteria
**Unjustified Claims**	Predicting	Question asks students to make (or select) a qualitative or non-calculable prediction about a given atom, molecule, reaction, or experiment without prompting for coupled reasoning.
	Comparing	Question asks students to make (or select) a ranking of atoms, molecules, subatomic particles, reactions, properties, or techniques without prompting for coupled reasoning.
	Evaluating Validity	Question asks students to categorize a given claim as either true or false without prompting for coupled reasoning.
	Classifying	Question asks students to provide (or select) an example of a(n) atom, molecule, reaction, or process given a particular property or scientific term without coupled reasoning OR classify a(n) atom, molecule, reaction, or process as a particular property or scientific term without coupled reasoning.
	Arguing Without Linkage	Question asks students to provide (or select) a claim and/or reasoning for a claim that is missing either a linkage between the evidence and reasoning or a coupled phenomenon.
**Mathematical Skills**	Setting Up Equations	Question asks students to set up (or select a set up for) a calculation or provide a mathematical relationship with no further use.
	Using Equations	Question asks students to perform a calculation and report (or select) the numerical value as the knowledge product with no interpretation.
	Counting	Question asks students to provide (or select) a tally of knowable (but not calculable) quantity of subatomic particles, bonds, atoms, molecules, significant figures, etc.
	Mathematical Thinking Lacking Phenomena	Question asks students to engage in analyzing & interpreting data and/or using mathematics & computational thinking; however, the question is not coupled to a phenomenon.
**Representational Skills**	Drawing/Labeling	Question asks students to draw (or select) a representation or label (or select) details of a representation (orbital, atom, molecule, graph, experiment, etc.) for a given event, observation, phenomenon, data, or scenario with no coupled reasoning and/or further use.
	Naming	Question asks students to provide (or select) the name of a molecule/compound given the formula/structure OR provide (or select) the formula/structure given the name of a molecule/compound with no further use.
	Writing Chemical Reactions	Question asks students to provide (or select) a balanced chemical equation, provide (or select) the coefficients necessary to balance a given reaction, OR provide (or select) products or reactants for a given reaction.
**Recalled Facts**	Recounting History	Question asks students to provide (or select) information about a historical event in chemistry with no further use or elaboration.
	Defining	Question asks students to provide (or select) the definitions for a scientific term OR provide (or select) the scientific term for a given definition.
	Describing	Question asks students to provide (or select) the details of a technique, experiment, or phenomenon for purely descriptive purposes.
**Not Content-Focused**	Miscellaneous	Question asks students for an affective or non-chemistry related response that is still worth credit (excluding those designated as extra credit).

Category 1: Unjustified Claims

Unjustified Claims	Predicting	Question asks students to make (or select) a qualitative or non-calculable prediction about a given atom, molecule, reaction, or experiment without prompting for coupled reasoning.
	Comparing	Question asks students to make (or select) a ranking of atoms, molecules, subatomic particles, reactions, properties, or techniques without prompting for coupled reasoning.
	Evaluating Validity	Question asks students to categorize a given claim as either true or false without prompting for coupled reasoning.
	Classifying	Question asks students to provide (or select) an example of a(n) atom, molecule, reaction, or process given a particular property or scientific term without coupled reasoning OR classify a(n) atom, molecule, reaction, or process as a particular property or scientific term without coupled reasoning.
	Arguing Without Linkage	Question asks students to provide (or select) a claim and/or reasoning for a claim that is missing either a linkage between the evidence and reasoning or a coupled phenomenon.

Code 11: Predicting

- Predicting the outcome of a phenomenon.
- Must be non-calculable and/or not based on an equation.
  - This means asking to predict the “sign” of a variable in an equation is not a prediction, but rather moved to the mathematical skills section (see code 21).
    - Generic descriptions about “increasing” or “decreasing” are okay.
    - This also applies for “trends” such as electronegativity, atomic radius, etc.
    - Ex:

> How would the following changes to the cell affect its potential delta E?
>
> Water is added to the anode: increases / decreases / stays the same
> Water is added to the cathode: increases / decreases / stays the same
> The electrodes are switched: increases / decreases / stays the same

  - Predicting the consequence of a perturbation on equilibrium DOES belong in this code when the question asks about a qualitative effect.
    - Ex:

> What would happen to the molar solubility of a solution of iron (III) nitrate when hydrochloric acid is added? (circle one)
>
> Little to no change / it will increase / it will decrease / need more info

    - Ex:

> Which of the following would lead to the generation of more carbon dioxide?
>
> $2CO (g) + O_2 (g) \rightleftharpoons 2CO_2 (g)$
>
> A. More O_2 (g) is added
> B. The temperature is decreased
> C. CO is removed
> D. All of these would lead to more CO_2
> E. None of these would lead to more CO_2

- Asking to predict a molecule's or solution's nature as "acidic, basic, or neutral" DOES belong in this code as it is a particular property.
    - Ex:

What will be the pH of the resulting solution after each of the following salts are dissolved in water:

NaBr	<7	~7	7<
LiOH	<7	~7	7<
NH_4Cl	<7	~7	7<

Code 12: Comparing

- When a question is asking for the student to choose the "most/least likely."
  - This criteria is more specifically for selected response questions where there are multiple provided answers and sometimes more than one applies, but there is a "best" option available.
    - Ex:

Circle the proton most likely to be deprotonated by hydroxide.

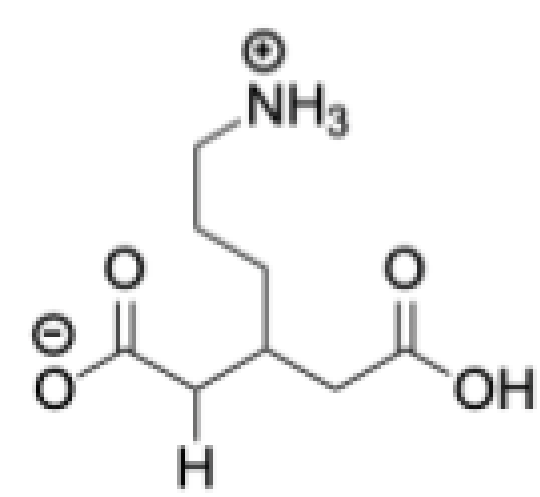

- When a question asks to rank two or more atoms, molecules, reactions based on properties such as:
  - Radius, electronegativity, polarity, intermolecular forces, redox potential, bond length, stability, reactivity, symmetry, acidity/basicity, mass, ionization energy, solubility, etc.
    - Ex:

List the following atoms in order of INCREASING atomic radius:

Ar F Ba C Br

Code 13: Evaluating Validity

- When the knowledge product is only "true" or "false" and does not meet the criteria for the scientific practice of "evaluating information"
  - These are the explicit T/F questions.
    - Ex:

> True or False
>
> The electronegativity of an atom increases as you move from left to right and bottom to top or the periodic table.

  - Some of the more implicit questions will ask about which statement is "true" or "correct" and if they vary by topic, it is not a comparison based on a single property (like code 12) and is just an evaluation of how true each statement is in regards to its own topic.
    - Ex:

> Which of the following statements regarding the combustion of octane is correct?
>
> A. The coefficient in front of oxygen in the balanced chemical equation is 24.
> B. The process is exothermic.
> C. The reactants are more polar than the products.
> D. Liquid water is a product of this reaction.

  - Identifying errors in particular representations, claims, etc. without needing to provide any justification.
    - Ex:

> Circle which of the following sets of quantum numbers is impossible.
>
> A. $n = 2, l = 2, m_l = 0, m_s = +½$
> B. $n = 1, l = 0, m_l = 1, m_s = +½$
> C. $n = 2, l = 2, m_l = -1, m_s = -½$
> D. $n = 3, l = 1, m_l = 1, m_s = +½$
> E. All of the above are possible

Code 14: Classifying

- When the question asks for an example of something like:
  - Scientific term/principle, type of reaction, atomic/molecular property, conjugate acid/base, etc.
    - Ex:

      Give an example of an alkali metal: _____

  - Atomic/molecular properties: geometry, hybridization, bond angles WHEN NOT COUPLED TO A PROVIDED REPRESENTATION (otherwise see code 31).
    - Ex:

      What is the molecular geometry around the central atom of PF_5?

- When the question asks to categorize a particular thing like:
  - Scientific term/principle, type of reaction, atomic/molecular property, etc.
    - Ex:

      H_2O (s) ⟶ H_2O (l) is what kind of phase change?

      A. Boiling
      B. Sublimation
      C. Melting
      D. Condensation

Code 15: Arguing Without Linkage

- Need all four to be considered the scientific practice of "developing and using models" or "constructing explanations and engaging in argument from evidence"
  - Phenomenon, event, or observation
    - Example when this is missing but has at least 2 of the others:

      Which has the larger radius and why?

      S or Na

  - Claim
  - Evidence/scientific principle that supports their claim
  - Reasoning that relates the evidence to the claim/phenomenon
    - Example when this is missing:

      Why does water have a higher boiling point than tetrahydrofuran (THF)?

      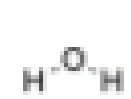

      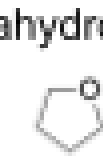

      A. Water experiences hydrogen bonding while THF only experiences dipole-dipole interactions.
      B. Water experiences dipole-dipole interactions while THF only experiences London dispersion forces.
      C. Water experiences London dispersion forces while THF only experiences hydrogen bonding.
      D. Water experiences hydrogen bonding while THF only experiences London dispersion forces.

Category 2: Mathematical Skills

**Mathematical Skills**	Setting Up Equations	Question asks students to set up (or select a set up for) a calculation or provide a mathematical relationship with no further use.
	Using Equations	Question asks students to perform a calculation and report (or select) the numerical value as the knowledge product with no interpretation.
	Counting	Question asks students to provide (or select) a tally of knowable (but not calculable) quantity of subatomic particles, bonds, atoms, molecules, significant figures, etc.
	Mathematical Thinking Lacking Phenomena	Question asks students to engage in analyzing & interpreting data and/or using mathematics & computational thinking; however, the question is not coupled to a phenomenon.

Code 21: Setting Up Equations

- This code includes predicting signs of variables in an equation
  - This includes entropy, enthalpy, Gibbs free energy, work, etc.
    - Ex:

> A non-spontaneous reaction is endothermic at 298 K. What is the sign for entropy?
>
> A. Positive
> B. Negative
> C. Need more information

- This code includes constructing an equation with variables without actually carrying it out
  - Equilibrium constant, entropy, enthalpy, Gibbs free energy, dissociation constant, rates (not rate law as that becomes either the scientific practice of analyzing and interpreting data or code 24), etc.
    - Ex:

> Write the equilibrium expression for the following reaction:
>
> $LiNO_3$ (*aq*) + NaBr (*aq*) $\rightleftharpoons$ LiBr (*aq*) + $NaNO_3$ (*aq*)

Code 22: Using Equations

- Applying a calculation to get a NUMERICAL knowledge product
  - Aside from explicit calculations, this can include bond order and oxidation number
    - Ex:

> Calculate the pH of a 2 M solution of acetic acid (pKa = 4.75) after 5 mL of .75 M NaOH is added.

Code 23: Counting

- This includes things that can be counted without the need of a calculation
    - This can include the number of valence electrons, the number of different bond types, significant figures, etc.
        - Ex:

How many sigma bonds and pi bonds are in the molecule below?

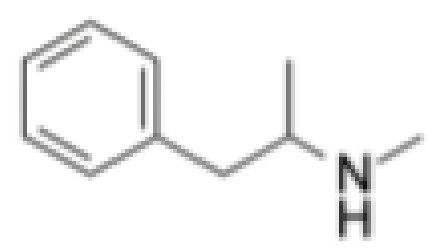

Sigma _____ Pi_____

Code 24: Mathematical Thinking Lacking Phenomena

- This code includes questions that lack the criteria of containing a phenomenon, event, or observation by our definition of phenomenon (see General Coding Notes) but would otherwise be considered the scientific practice of analyzing and interpreting data or using mathematics and computational thinking.
    - These questions often provide hypothetical reactions and/or data
        - Ex:

Determine the rate law for the following reaction:

A + BC DE

[A]	[BC]	Rate
1 M	1 M	3.00
2 M	1 M	9.00
1 M	3 M	3.00

Category 3: Representational Skills

**Representational Skills**	Drawing/Labeling	Question asks students to draw (or select) a representation or label (or select) details of a representation (orbital, atom, molecule, graph, experiment, etc.) for a given event, observation, phenomenon, data, or scenario with no coupled reasoning and/or further use.
	Naming	Question asks students to provide (or select) the name of a molecule/compound given the formula/structure OR provide (or select) the formula/structure given the name of a molecule/compound with no further use.
	Writing Chemical Reactions	Question asks students to provide (or select) a balanced chemical equation OR provide (or select) the coefficients necessary to balance a given reaction.

Code 31: Drawing/Labeling

- This code is used for questions that are directly coupled to an explicit representation
    - Some selected response questions will prompt the student to draw a Lewis structure and then ask for a claim about the Lewis structure; as the Lewis structure is not the official knowledge product and the representation was not provided, these questions can not be considered for labeling representations.
        - Ex:

Q1a. Provide the Lewis structure for sulfuric acid:

Code 31

Q1b. How many sigma bonds are there?

Code 23

    - This code includes drawing generic shapes on a graph, indicating something on a representation (not an extrapolation of the data), Molecular Orbital diagrams, electron configurations, atomic symbols (and all of their parts: electrons, neutrons, and protons), adding something to an existing representation (such as finishing a Lewis structure with lone pairs and/or bonds), Lewis structures, depictions of orbitals, full quantum numbers for an electron, labeling functional groups, etc.
        - Ex:

Fill in the MO diagram for O_2.

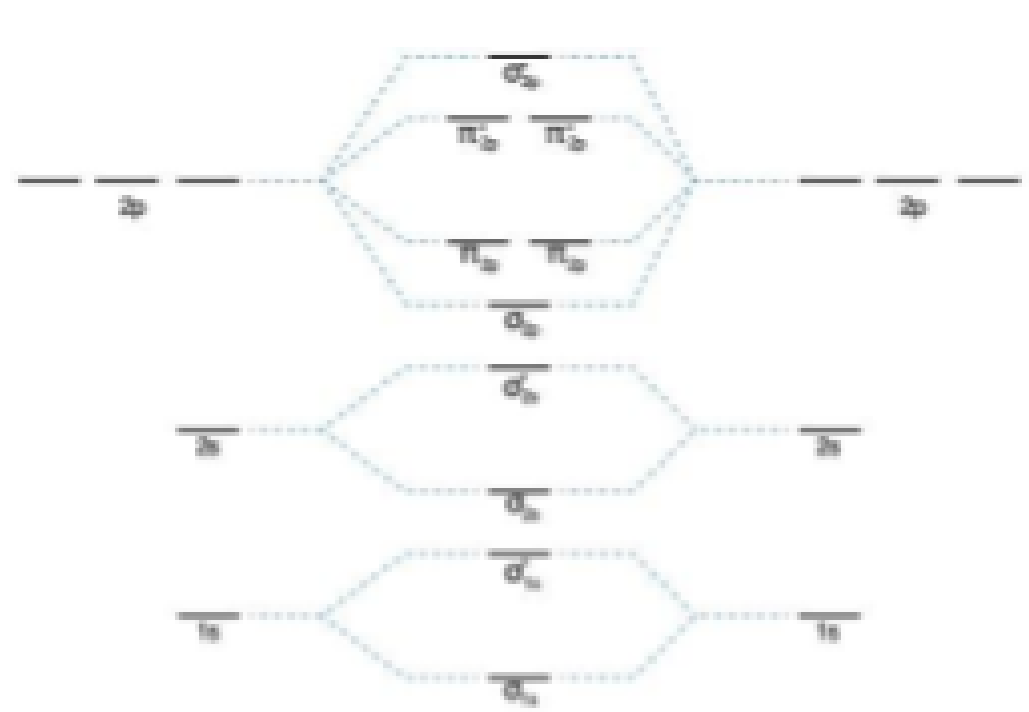

Code 32: Naming

- Needs to be a direct translation from provided structure, name, or formula
  - Asking the student to perform another type of task and report the knowledge product as a name is NOT included here but coded with respect to the actual task prompted
    - Ex:

> Provide the name for the following compound:
>
> $Fe_2(SO_4)_3$ ______________________

Code 33: Writing Chemical Reactions

- This code includes the explicit writing of a chemical reaction (or selection of it) as well as balancing reactions that are provided sans their coefficients.
  - This can include predicting the products of a reaction, half reactions, nuclear decay reactions, net and ionic reactions, etc.
  - This is NOT when asked to select the reaction that is an example of _______ (see code 14 for these)
    - Ex:

> Write the balanced chemical equation for the acid/base reaction that happens between NaOH and HCl:

- Ex:

> What is the coefficient in front of water in the balanced chemical reaction when octane combusts?
>
> $\_\_C_8H_{18} + \_\_O_2 \rightleftharpoons \_\_H_2O + \_\_CO_2$
>
> ↑

Category 4: Recalled Facts

**Recalled Facts**	Recounting History	Question asks students to provide (or select) information about a historical event in chemistry with no further use or elaboration.
	Defining	Question asks students to provide (or select) the definitions for a scientific term OR provide (or select) the scientific term for a given definition.
	Describing	Question asks students to provide (or select) the details of a technique, experiment, or phenomenon for purely descriptive purposes.

Code 41: Recounting History

- This code can be (inter)nationally known events as well as events that happened in the coupled laboratory part of the course.
    - The "who did what" and "what happened at this moment in time" type of questions.
        - Ex:

When you did the solubility lab, what was the precipitate that formed?

        - Ex:

Match the following scientist with their contribution to chemistry:

A. Provided the framework for the periodic table
B. Discovered the electron
C. Discovered and named the atomic nucleus and the proton
D. Provided the first evidence that supported Bohr's atomic model aside from the hydrogen emission spectrum
E. Introduced the atomic theory

___ Ernest Rutherford ___ J.J. Thomson ___ John Dalton ___ Dmitri Mendeleev ___ Henry Moseley

Code 42: Defining

- Needs to be a direct prompt for a canonical definition of a scientific term/principle
    - This does NOT include questions asking for examples of a particular scientific term/principle (see code 14)
        - Ex:

Which is the best definition for chemical equilibrium?

A. When the rate of the reverse reaction is slower than the rate of the forward reaction
B. When the concentration of reactants is equal to the concentration of products
C. When the rate of the reverse reaction is equal to the rate of the forward reaction
D. When the concentration of products is greater than the concentration of reactants

Code 43: Describing

- These are not definitions, but can include general descriptors of scientific laws/theories
    - Tenets of particular theories, what information they can provide, etc.
        - Ex:

> What are the three laws of thermodynamics? What do they tell us?

- Descriptions of an experiment or technique to achieve a particular type of information
    - The “how would you do this” type of question that does not meet the criteria for the scientific practice of “planning investigations”
        - Ex:

> Describe what a titration does and the steps for how to do one step by step (NO calculations).

Category 5: Not Content-Focused

**Not Content-Focused**	Miscellaneous	Question asks students for an affective or non-chemistry related response that is still worth credit (excluding those designated as extra credit).

Code 51: Miscellaneous

- These questions are typically subjectively affective in nature and/or do not require the application of chemistry course-specific content to answer
  - Asking for opinions, jokes, syllabus information, etc.
    - Ex:

      What do you call CH_2O?

      A. Ocean water
      B. This was a bad joke
      C. Both A and B
    - Ex:

      What percentage of course points are allotted to assessments?

      A. 35%
      B. 40%
      C. 55%
      D. 100%
    - Ex:

      What was your favorite unit this semester and why?
