# Supplementary material for: What Counts in General Chemistry? An Exploratory Look at What Was Emphasized and Rewarded on General Chemistry Exams Across the United States
Source: J Chem Educ. 2026 Jul 5;103(8):4086–98. doi: 10.1021/acs.jchemed.6c00309 (PMC13472132; doi:10.1021/acs.jchemed.6c00309)
Supplement: Supplementary file 2 [file ed6c00309_si_002.pdf]

Supporting Information

What Counts in General Chemistry? An Exploratory Look at What Was Emphasized and Rewarded on General Chemistry Exams Across the United States

Nicole C. Greco, Niall J. Ellias, and Ryan L. Stowe*

*Correspondence to rstowe@chem.wisc.edu

Department of Chemistry, University of Wisconsin – Madison, 1101 University Avenue, Madison, Wisconsin 53706-1322, United States

Subcategory Breakdown: R1

Subcategory	Frequency	Percentage
Predicting	107	5.17%
Comparing	269	13.00%
Evaluating Validity	153	7.39%
Classifying	238	11.50%
Arguing Without Linkage	74	3.57%
Setting Up Equations	114	5.51%
Using Equations	554	26.76%
Counting	70	3.38%
Mathematical Thinking Lacking A Phenomenon	70	3.38%
Drawing/Labeling	301	14.54%
Naming	11	0.53%
Writing Chemical Reactions	48	2.32%
Recounting History	7	0.34%
Defining	20	0.97%
Describing	21	1.01%
Miscellaneous	13	0.63%
**Total Tasks**	**2070**	**100%**

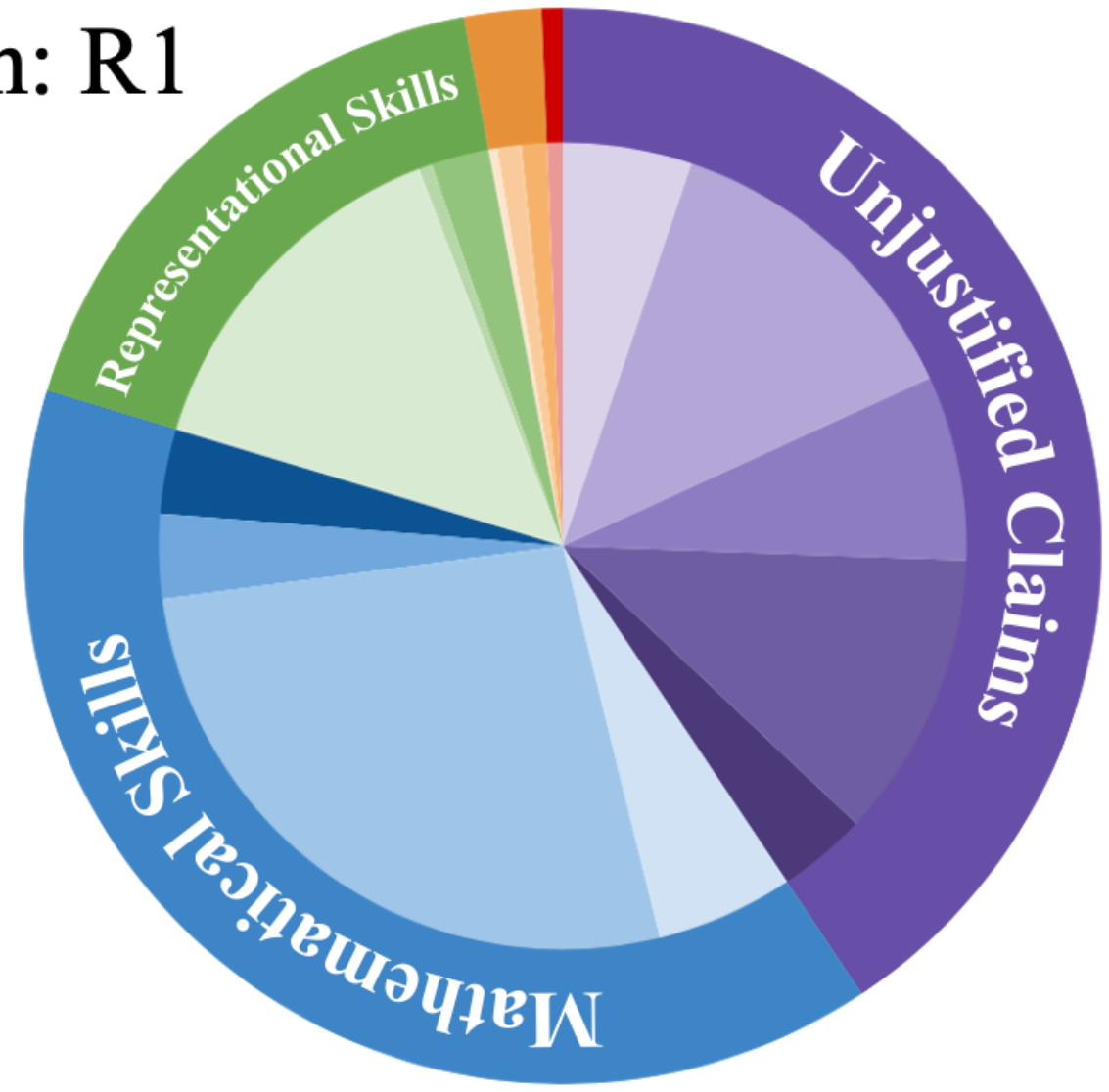

18 instructors (16 institutions), 76 exams

Subcategory Breakdown: R2

Subcategory	Frequency	Percentage
Predicting	28	4.99%
Comparing	45	8.02%
Evaluating Validity	5	0.89%
Classifying	88	15.69%
Arguing Without Linkage	6	1.07%
Setting Up Equations	15	2.67%
Using Equations	159	28.34%
Counting	37	6.60%
Mathematical Thinking Lacking A Phenomenon	15	2.67%
Drawing/Labeling	80	14.26%
Naming	24	4.28%
Writing Chemical Reactions	37	6.60%
Recounting History	2	0.36%
Defining	9	1.60%
Describing	11	1.96%
Miscellaneous	0	0.00%
**Total Tasks**	**561**	**100%**

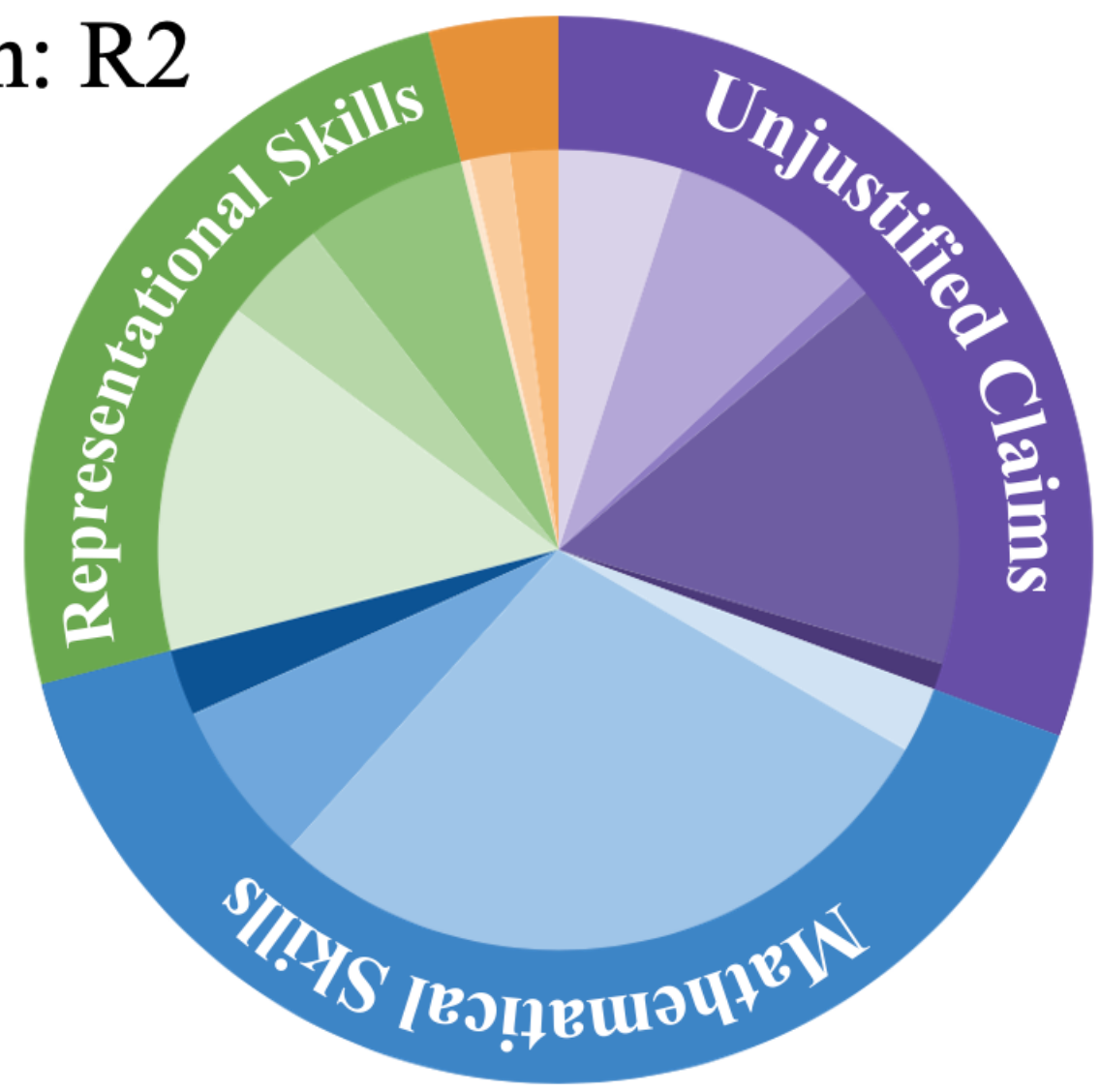

5 instructors (5 institutions), 22 exams

Subcategory Breakdown: PUI

Subcategory	Frequency	Percentage
Predicting	152	3.76%
Comparing	421	10.42%
Evaluating Validity	95	2.35%
Classifying	650	16.09%
Arguing Without Linkage	75	1.86%
Setting Up Equations	171	4.23%
Using Equations	1116	27.62%
Counting	153	3.79%
Mathematical Thinking Lacking A Phenomenon	64	1.59%
Drawing/Labeling	623	15.42%
Naming	145	3.59%
Writing Chemical Reactions	175	4.33%
Recounting History	17	0.42%
Defining	104	2.58%
Describing	72	1.78%
Miscellaneous	7	0.17%
**Total Tasks**	**4040**	**100%**

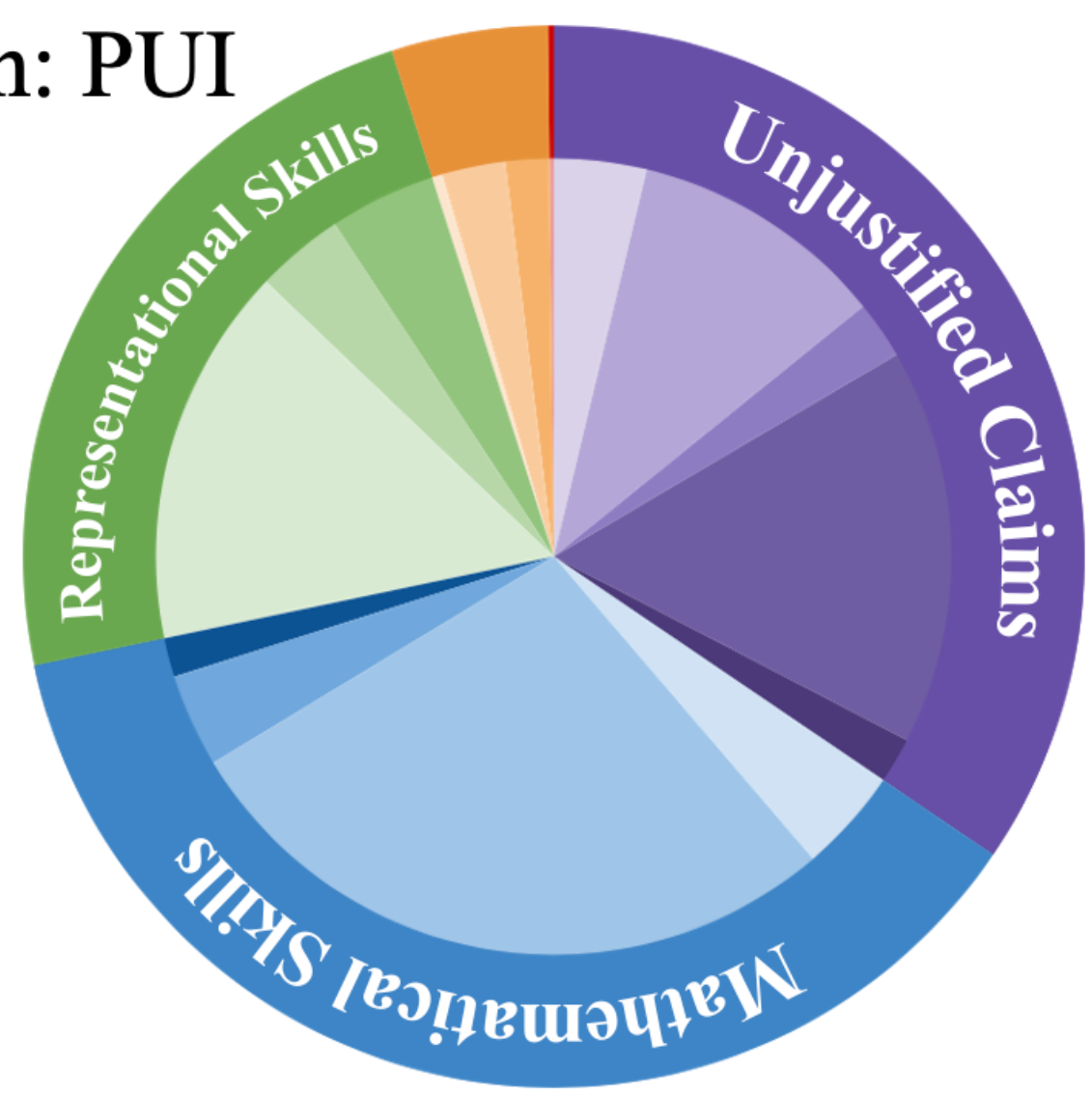

37 instructors (36 institutions), 137 exams
